# A new molecular subclassification and *in silico* predictions for diagnosis and prognosis of papillary thyroid cancer by alternative splicing profile

**DOI:** 10.3389/fphar.2023.1119789

**Published:** 2023-03-06

**Authors:** Haiyan Li, Hao Lan, Menglong Li, Xuemei Pu, Yanzhi Guo

**Affiliations:** College of Chemistry, Sichuan University, Chengdu, China

**Keywords:** alternative splicing (AS), papillary thyroid cancer (PTC), subclassification, *in silico* prediction, diagnosis, prognosis

## Abstract

**Introduction:** Papillary thyroid cancer (PTC) is the most common endocrine malignancy. However, different PTC variants reveal high heterogeneity at histological, cytological, molecular and clinicopathological levels, which complicates the precise diagnosis and management of PTC. Alternative splicing (AS) has been reported to be potential cancer biomarkers and therapeutic targets.

**Method:** Here, we aim to find a more sophisticated molecular subclassification and characterization for PTC by integrating AS profiling. Based on six differentially expressed alternative splicing (DEAS) events, a new molecular subclassification was proposed to reclassify PTC into three new groups named as Cluster0, Cluster1 and Cluster2 respectively.

**Results:** An *in silico* prediction was performed for accurate recognition of new groups with the average accuracy of 91.2%. Moreover, series of analyses were implemented to explore the differences of clinicopathology, molecular and immune characteristics across them. It suggests that there are remarkable differences among them, but Cluster2 was characterized by poor prognosis, higher immune heterogeneity and more sensitive to anti-PD1 therapy. The splicing correlation networks proved the complicated regulation relationships between AS events and splicing factors (SFs). An independent prognostic indicator for PTC overall survival (OS) was established. Finally, three compounds (orantinib, tyrphostin-AG-1295 and AG-370) were discovered to be the potential therapeutic agents.

**Discussion:** Overall, the six DEAS events are not only potential biomarkers for precise diagnosis of PTC, but also the probable prognostic predictors. This research would be expected to highlight the effect of AS events on PTC characterization and also provide new insights into refining precise subclassification and improving medical therapy for PTC patients.

## 1 Introduction

Papillary thyroid cancer (PTC) is the most common histological type of thyroid cancer (Fagin and Wells, 2016; Wang et al., 2020). Its precise diagnosis and prognosis have all-important clinical significance. It is commonly classified into three histological variants, including classical papillary thyroid cancer (CPTC), follicular papillary thyroid cancer (FPTC) and tall cell papillary thyroid cancer (TCPTC), which collectively account for the vast majority of PTCs (Shi et al., 2016). CPTC is usually characterized by papillary architecture containing typical nuclear features of chromatin pallor, nuclear enlargement, grooves and pseudoinclusions (Pusztaszeri and Auger, 2017). TCPTC commonly occurs at advanced stages with a higher incidence of local recurrence and distant metastasis (Lloyd et al., 2011). FPTC, characterized by nuclear of classical PTC and follicular cell growth patterns, has been shown to be indolent with virtually no metastatic or recurrence potential so that the encapsulated/well-demarcated, non-invasive FPTC have been renamed as non-invasive follicular thyroid neoplasm with papillary-like nuclear features or “NIFTP” (Shi et al., 2016; Tallini et al., 2016). Different variants differ at histological, cytological, molecular and clinicopathological levels, which complicates the precise diagnosis and management of PTC. So deciphering PTC subtypes plays a vital role in predicting patients’ prognosis, avoiding misdiagnosis and inadequate/aggressive therapy.

Except the histopathological variants, PTC also has been divided into different subtypes based on different data: five based on mRNA expression, six on miRNA expression, four on DNA methylation and four on protein expression, which might better inform the management of PTC (Agrawal et al., 2014). Nevertheless, PTC is a heterogeneous neoplasm from both morphological and molecular perspectives. Therefore, more refined molecular classifications of PTC and the identification of new markers associated with PTC subtypes is of great practical significance for precise diagnosis, surgical and medical therapy of PTC patients.

Alternative splicing (AS) refers to the process that a pre-mRNA can be processed into different mature mRNA molecules in which an exon/intron could be differentially included/excluded by the choice of specific AS sites (Liu and Rabadan, 2021). It has been estimated that pre-mRNA splicing is essential for the expressions of more than 95% of all human genes (Bonnal et al., 2020). Recently, the roles of AS in the development of tumors have been revealed that AS is involved in the processes of proliferation, differentiation and apoptosis *via* regulating the alternative expression of many oncogenes or antioncogenes, since it is widely deregulated in multiple cancer types (Sciarrillo et al., 2020; Zhang et al., 2021b). Besides, increasing evidences have indicated that AS could be the potential biomarkers for the diagnosis, prognosis, therapy and monitoring of cancer patients (Bonnal et al., 2020). Moreover, AS shows heterogeneity among different cancers subtypes and AS events have been utilized for the identification of cancer subtypes. So deep insights into AS events and their potential for cancer precise diagnosis and prognosis are vital for better understanding the potential molecular mechanisms underlying PTC subtypes.

To provide more perspectives for PTC molecular classifications and to explore the landscape of AS events and their potential values for precise diagnosis and prognosis in PTC, we proposed a new molecular subclassification and characterization for PTC by integrating AS profiling. Then *in silico* predictions for precise diagnosis and prognosis of PTC were performed using machine-learning methods. A comprehensive analysis was conducted to detect the differences of clinicopathology, molecular and immune characteristics among the new subgroups. At last, the potential drug screening for PTC was performed for improving the clinical treatment of patients with high-risk or advanced stages.

## 2 Materials and methods

### 2.1 Data collection and pre-processing

Splicing data for PTC patients were downloaded from TCGA SpliceSeq database (https://bioinformatics.mdanderson.org/TCGASpliceSeq/index.jsp) with default settings. SpliceSeq database evaluates seven types of splicing events, including alternate acceptor site (AA), alternate donor site (AD), alternate promoter (AP), alternate terminator (AT), exon skip (ES), mutually exclusive exons (ME) and retained intron (RI) respectively. The PSI value ranging from 0 to 1 indicates the efficiency of a certain splicing process. Here it was used for quantification of AS events. The clinical data, gene expression and somatic mutation annotation files were from TCGA (http://portal.gdc.cancer.gov/). Only patients were included in our analysis with the following information: 1) PTC variants, 2) corresponding mRNA expression and AS PSI values, 3) relatively complete clinical information on sex, age, stage and survival, and 4) the follow-up periods of more than 90 days. Molecular signatures in gene set collection of KEGG and REACTOME in curated canonical pathways (C2) and gene ontology (GO) terms (C5) were downloaded from the molecular signatures database (MSigDB) (https://www.gsea-msigdb.org/gsea/msigdb/index.jsp). The splicing factors (SFs) gene list was collected from the SpliceAid 2 (http://www.introni.it/splicing.html) and displayed in Supplementary File S1; Supplementary Table S1.

The average PSI value of each AS event in all samples was used to interpolate the missing values and minimize the possible bias caused by the missing values. To generate a reliable set of AS events, a strict filtering was conducted with average PSI value≧0.05 and standard deviation of PSI value≧0.1. Finally, 422 PTC patients with complete AS PSI values, gene expression data and corresponding clinical information were achieved for the further analyses, including 312 CPTC samples, 96 FPTC and 34 TCPTC, respectively. Moreover, to accurately describe the AS events, the unique annotation of each AS was named by combing the gene symbol, the ID number in the SpliceSeq database and the splicing type, for example, DCN_23655_AT. Details of the whole dataset can be seen in Supplementary File S2; Supplementary Table S1.

### 2.2 Identification of DEAS and PTC variants classification

To identify the DEAS between CPTC and FPTC, CPTC and TCPTC, as well as FPTC and TCPTC, log2FC and Wilcoxon test were conducted. AS events with an absolute value of |log2FC| ≧1 and *p*-value<0.05 were considered as statistically significant. Only DEAS events that are significantly differentially expressed in all three variants are selected for further analyses.

### 2.3 Clustering analysis and *in silico* training for model construction

Here, k-means clustering algorithm was performed on the PTC cohorts based on the identified DEAS events and principle component analysis (PCA) was used to evaluate the difference among different PTC variants as well as to validate the clustering result. For the construction of the classifier model for distinguishing the clustering groups, the commonly used machine-learning algorithm, support vector machine (SVM) was selected. Using the identified DEAS events as the feature inputs, a linear SVM classifier was trained to classify three clustering groups by “kernlab” package. We performed 10-fold cross-validation to train and test the classifier. To verify the robustness of the model, we executed 100 times of 10-fold cross-validation. So the model training and testing were repeated 100 times and the performance of the classification model was assessed by averaging the accuracy (ACC), Precision, Recall and F1 score of 100 times. The following are the equations of four evaluation parameters:

$$ACC=\frac{TP+TN}{TP+FP+TN+FN}$$ (1)

$$Precision=\frac{TP}{TP+FP}$$ (2)

$$Recall=\frac{TP}{TP+FN}$$ (3)

$$F1=\frac{2*Precision*Recall}{Precision+Recall}$$ (4)

Where TP, FP, TN and FN are the true positive, false positive, true negative and false negative, respectively.

### 2.4 Differences analysis

A comprehensive difference analysis was performed to assess the rationality of the newly classified clusters. In our research, we considered clinicopathological variables (T stage, N stage, M stage and TNM stage), immune landscape (immune score, stromal score, immune checkpoint inhibitors expression, immune cell infiltration), survival status (SS), tumor purity, tumor mutational burden (TMB) and immunotherapy response.

ESTIMATE is a widely used method that can deduce the fraction of immune and stromal cells in tumor samples using the gene expression profile (Yoshihara et al., 2013). Tumor purity reveals the percentage of malignant cells in a solid tumor sample. Immune scores, stromal scores and tumor purity of each PTC patient were calculated by ESTIMATE algorithm. CIBERSORT was performed to evaluate the proportions of all 22 immune cells based on the gene expression profile by running CIBERSORT script from the website (http://rdrr.io/github/singha53/amritr/src/R/supportFunc_cibersort.R). Moreover, we compared the expressions of five immune checkpoints including CTLA4, TIGIT, HAVR2, PDCD1 and CD274 among clustering groups. The “maftools” package was used to obtain TMB of all PTC samples (Mayakonda et al., 2018). To compare the gene expression matrices between our three clusters and those cancer patients treated with immune checkpoint blockade (ICB) therapy, the subclass mapping method (SubMap) (Hoshida et al., 2007) was conducted. Here, transcriptomic data of 65 cancer patients treated by anti-PD1 therapy were used (Prat et al., 2017) and details about 65 patients are shown in Supplementary File S2 Supplementary Table S2. This step was implemented on SubMap module of the GenePattern website (http://genepattern.broadinstitute.org/) with default parameters setting. Gene set variation analysis (GSVA) was implemented by “clusterProfiler” package to acquire the GSVA scores of biological pathways and GO terms of each PTC patient (Hänzelmann et al., 2013). The “limma” package was used to investigate significant differentially pathways and GO terms and those with an adjusted *p*-value <0.05 were considered as statistically significant (Ritchie et al., 2015).

At last, the splicing-related networks for AS events and SFs were also constructed for discovering the regulation relationships between AS and SFs of each clustering group. For splicing factor (SF)-AS regulatory network construction, a list of 71 splicing factor genes was achieved from the SpliceAid two database, and the mRNA expression profile of SF genes was downloaded from TCGA database. To screen out the survival-related AS events in different clustering groups, univariate Cox regression analyses were performed and AS events with *p*-value <0.05 were identified as overall survival (OS)-related AS events. Then, Spearman correlation method was used to calculate the correlation coefficients between PSI values of OS-related AS events and the expression values of SF genes. Finally, the splicing-related network for AS events and SFs, named as SF-AS regulatory network was built and drawn by the software of Cytoscape (version 3.7.1).

### 2.5 Establishment of DEAS-based prognostic model and survival analysis

To evaluate the prognostic value of the identified DEAS events, we performed Kaplan-Meier curve analysis to estimate the relationships between the identified DEAS events and OS. Based on PSI values, DEAS events with *p*-value<0.05 were considered as prognostic AS events. In addition, The six DEAS events were entered into the step-wise multivariate Cox regression analysis using R package “survminer” to select the key DEAS events with great prognostic values. And the DEAS events screened in the multivariate Cox regression were used to build the PTC OS-associated signature. The signature is obtained by the following formula:

$$RiskScore=\overset{n}{\underset{i=1}{\sum }}\beta_{i}*PSI_{i}$$ (5)

Where *β*_*i*_ is the regression coefficient, *PSI*_*i*_ is the PSI value of the corresponding DEAS event. PTC patients would be divided into high- and low-risk groups by the cutoff value that is determined by the X-tile, and the Kaplan-Meier curve was plotted to show the different prognoses. Besides, area under curve (AUC) from the receiver operating characteristic (ROC) curves of 1, 3 and 5 years and concordance index (C-index) were calculated to estimate the prognostic performance of the signature. To determine whether the prognostic signature is independent of clinical factors, the signature and confounding clinicopathological variables including age, gender and TNM stage were analyzed using univariate and multivariate Cox regression analyses.

### 2.6 CMap analysis

The connectivity map (CMap) online tool (https://clue.io) was used to predict the effect of drugs on the specific gene expression patterns in tumors. Differentially expressed analysis was implemented to collect the top 150 up-expressed genes in the high-risk cluster. Then, the top 150 up-regulated genes were uploaded into the CMap online tool. The matches between genes and chemicals were assessed by scores from -100 to 100. A positive score implicates a stimulative effect of compound on the query signatures, while a negative score indicates a repressed effect of a compound on the query signatures. Bioactive chemicals with a negative score can be candidate drugs for the treatment of patients.

## 3 Results

### 3.1 Overview of AS events in PTC cohort

**FIGURE 1:**
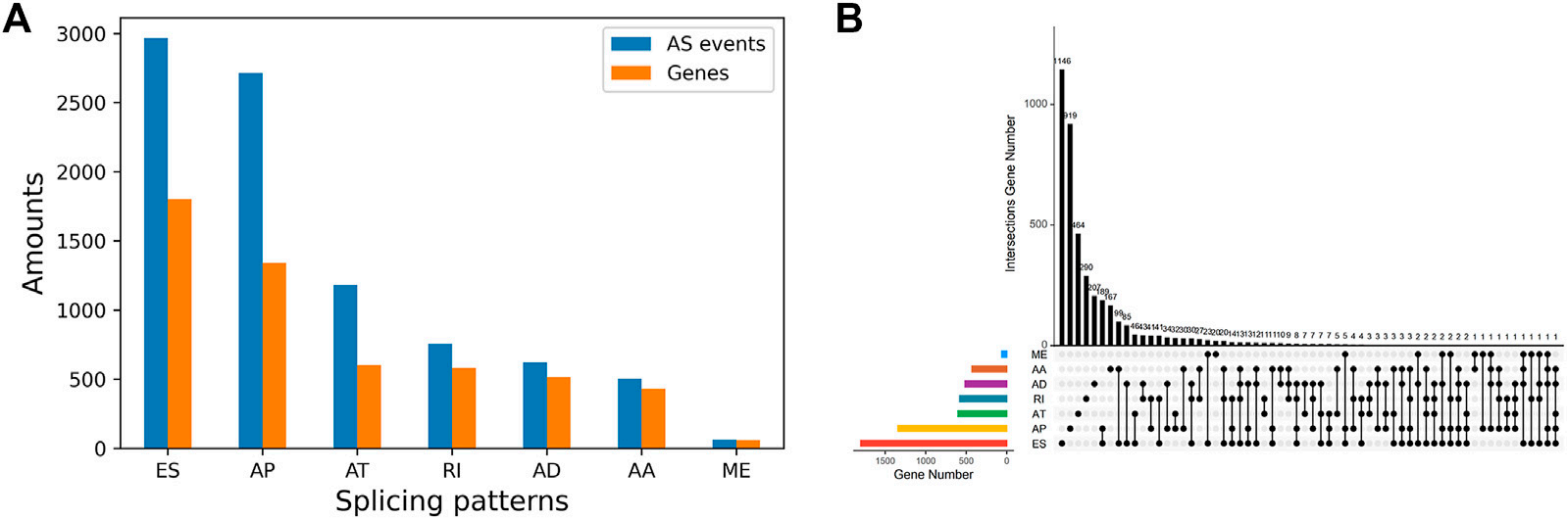
Summarization of AS events in PTC Cohort **(A)** The number of AS events and their parental genes **(B)** The upset plot of the intersection among seven types of AS events.

The splicing data of 442 PTC patients from TCGA SpliceSeq database were analyzed. Then a total of 8833 AS events were detected from 4,143 genes using rigorous filtering criteria. Figure 1A shows the distributions of all AS events and genes with seven different splicing patterns, including 503 AA in 429 genes, 620 AD in 514 genes, 2741 AP in 1,341 genes, 1183 AT in 601 genes, 2966 ES in 1801 genes, 64 ME in 61 genes and 756 RI in 581 genes, respectively. Besides, the UpSetR plot in Figure 1B shows that a high proportion of genes may have two or more different splicing patterns and at least five different AS events could occur in one single gene, suggesting the complicated regulation relationships between genes and AS events.

### 3.2 Identification of DEAS and PTC variants classification

To identify DEAS events in PTC variants, the PSI values of CPTC, FPTC and TCPTC were analyzed. So 59 DEAS events were obtained between CPTC and FPTC, 17 between CPTC and TCPTC, and 199 FPTC and TCPTC respectively. The details are shown in Supplementary File S1; Supplementary Table S2 and Supplementary Figures S1A–C. By acquiring the intersections in Supplementary File S1; Supplementary Figure S1D, six DEAS events were identified that are differentially expressed in all three PTC variants, including NNMT_18817_AP, DCN_23655_AT, TUBB3_38175_ES, KIAA1217_10995_AP, COL14A1_85015_AP and RCAN2_76415_AP.

To verify the distinguishing ability of the identified DEAS events for CPTC, FPTC and TCPTC, PCA analysis were performed, but Supplementary File S1; Supplementary Figure S2 shows that three variants cannot be separated from each other. Then we tried to classify them by constructing a DEAS-based SVM classifier. However, the model training result also gives the overall prediction accuracy with 0.505 and F1 with 0.483. So we can conclude that the six identified DEAS events cannot be the classifying biomarkers for the current three variants.

### 3.3 DEAS-based new subclassification and *in silico* prediction for new clusters

**FIGURE 2:**
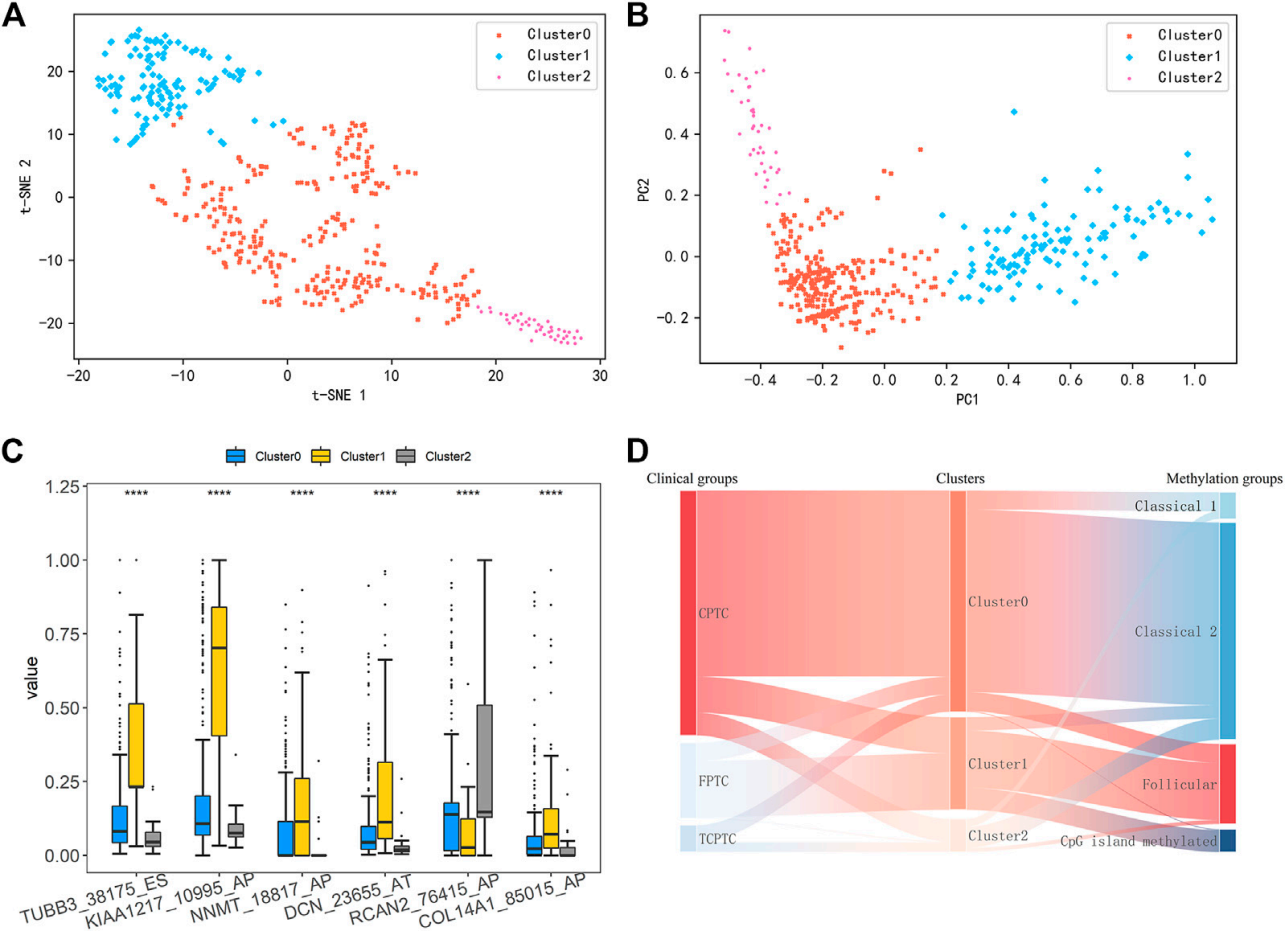
**(A)** Results of K-means clustering analysis was visualized by t-SNE algorithm **(B)** PCA of three distinct clusters was shown in a scatter plot **(C)** Differential analysis on PSI values of six DEAS events among three clustering groups (****: *p* < 0.0001) **(D)** Sankey Diagram showing comparisons between our clusters, PTC variants and subtypes based on DNA promoter methylation.

Considering that there are no effective differences among the current PTC variants, all PTC patients were re-clustered using k-means clustering analysis based on the six identified DEAS events. The clustering result was visualized by t-SNE algorithm. As shown in Figure 2A, all PTC patients were clearly clustered into three groups, including 282 samples in Cluster0, 118 in Cluster1 and 42 in Cluster2, respectively. Details about the clinical and demographic data of PTC patients in Clusters 0, 1 and two are shown in the Supplementary File S2; Supplementary Table S3. Consistent with the clustering result, PCA analysis in Figure 2B also reveals virtual differences among Cluster0, Cluster1 and Cluster2. Consequently, we also validated the differences of these DEAS events in the three new clustering groups. The box plot in Figure 2C shows that the six DEAS events exhibit the significant differences across three clusters. The detailed information is shown in Supplementary File S1; Supplementary Table S3. So we can see that all PTC patients could be newly classified into additional three groups based on the six identified DEAS events, which also indicates that these DEAS could be the potential biomarkers for PTC subclassification and characterization of PTC.

Here, we compared our three clusters with the current histological variants and the previously proposed four DNA promoter methylation-based clusters by Agrawal et al. (Agrawal et al., 2014). As shown in Figure 2D, most of patients belonging to Cluster0 show an enrichment of CPTC, and Classical 1 and two of DNA promoter methylation-based clusters. In addition, clusters annotated as Follicular and CpG island methylated were mostly assigned to Cluster1. Those within Cluster2 were almost observed in Classical two and Classical 1. These findings manifest that Cluster0 and Cluster2 share obvious similarity with Classical 1 and Classical 2, but Cluster1 is similar with Follicular and CpG island methylated. Overall, this subclassification is novel and different from the current available subtypes.

### 3.4 *In silico* prediction for new clusters

**FIGURE 3:**
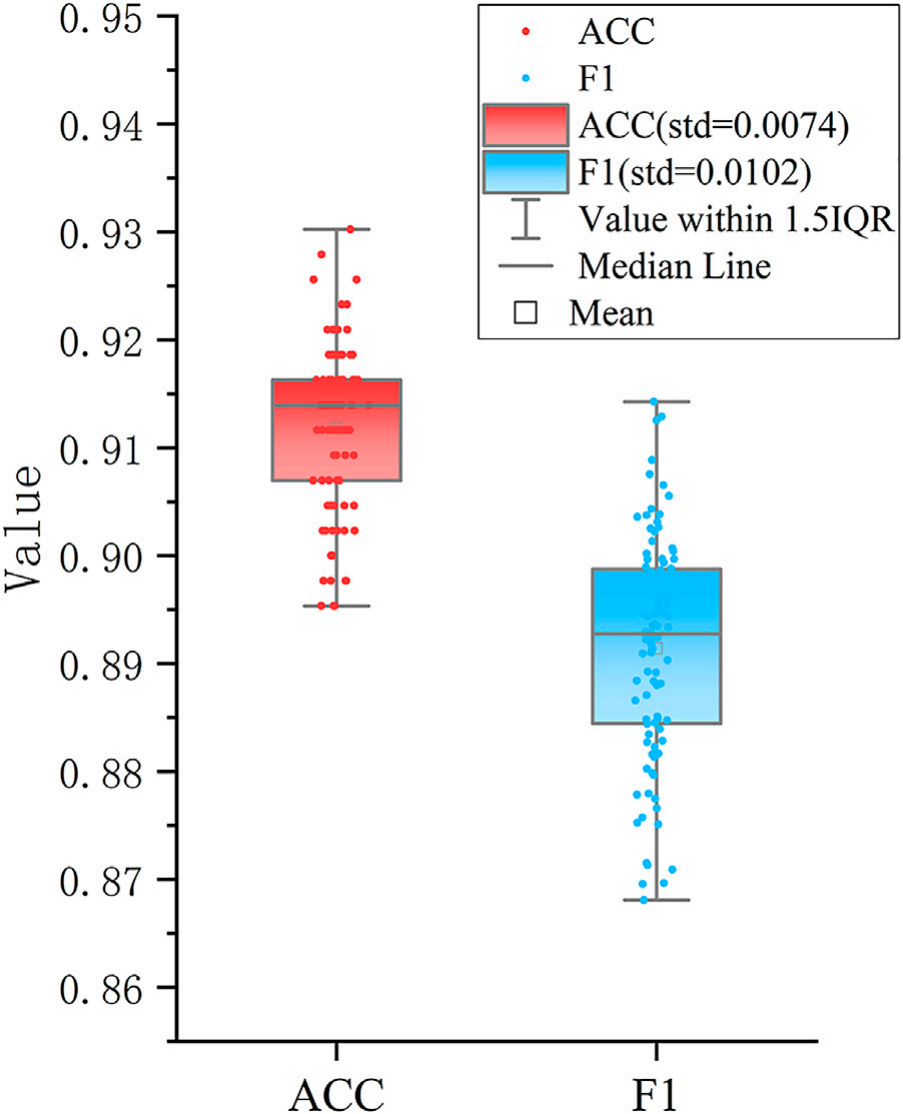
Performance of the SVM based classifier. The boxplots show the distributions of ACC and F1 values of 100 different testing sets produced by performing 100 round 10-fold cross-validations.

Furthermore, a classification model was established for distinguishing the three new groups using these six DEAS events as feature input. To build an effective triple-class recognition model, we selected SVM to train the data and the process of the model construction and testing was repeated 100 times by performing 100-round 10-fold cross-validations. The average prediction performance is shown in Table 1. Overall, the model gives promising prediction performance with the ACC of 91.2%. Moreover, each cluster also yields satisfactory result with Recall of 0.879, 0.998 and 0.981 for Cluster0, Cluster1 and Cluster2 respectively. The distributions of ACC and F1 values in Figure 3 indicate that 100 models all give comparable performance, indicating that the model is robust although it simultaneously predicts the three clusters.

**TABLE 1:** Classification performance of the model for identifying three clusters.

Clusters	ACC	Precision	Recall	F1
Cluster0	_	0.993	0.879	0.934
Cluster1	_	0.856	0.998	0.929
Cluster2	_	0.737	0.981	0.830
Overall	0.912 ± 0.007	0.862 ± 0.012	0.948 ± 0.009	0.891 ± 0.011

### 3.5 Differences of clinicopathological, immune and molecular features across the DEAS-based clusters

**FIGURE 4:**
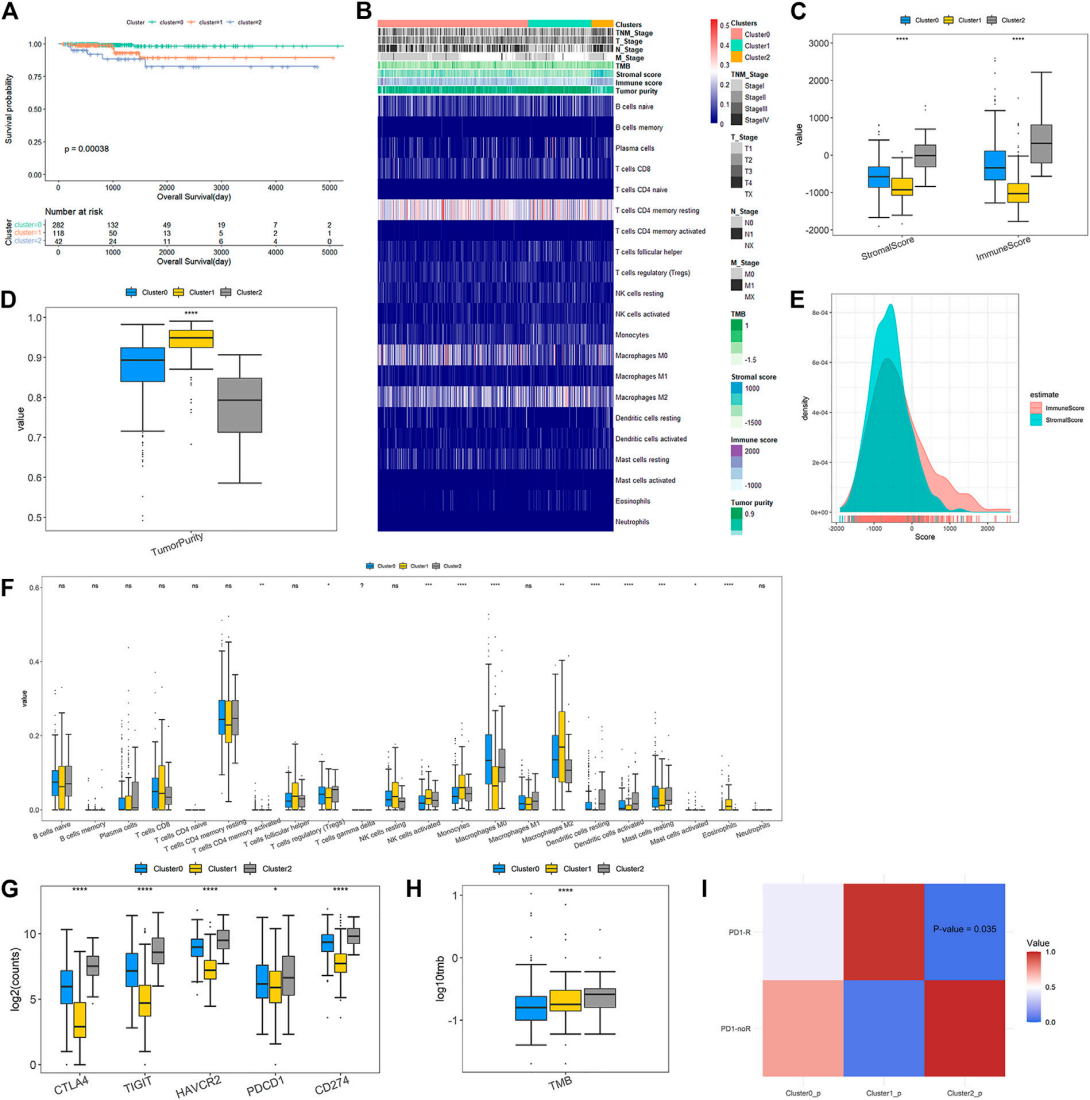
Clinicopathological characteristics and immune microenvironment features across DEAS-based clusters **(A)** Kaplan-Meier survival analysis of patients within three clustering subtypes of OS **(B)** A total of 442 PTC patients ordered by distinct clusters with annotations with cliniaopathological characteristics and immune features were visualized in a matrix heatmap **(C)** Immune and stromal scores of each DEAS-based cluster **(D)** Tumor purity of three clusters **(E)** Density curve of immune and stromal scores of all PTC patients **(F)** Comparisons on the proportions of immune infiltrating cells between three clusters **(G)** Expressions of immune checkpoints across the three clusters **(H)** Tumor mutation burden of three clusters **(I)** Responses to anti-PD1 therapy. The color in the cells represent the *p* values.

The comprehensive difference analysis was performed on our new subclassfication in terms of clinical, immune and molecular characteristics. Firstly, Kaplan-Meier curve analysis was performed to assess the relationships between clusters and OS. From Figure 4A, we can observe the significant prognosis difference across three clusters with *p*-value of 0.00038. The PTC patients belonging to Cluster2 have the worse prognosis than those within Cluster0 and Cluster1. Furthermore, a higher proportion of patients with stage III and IV, T3/T4 and N1 are observed in Cluster2 compared with Cluster0 and Cluster1 (Figure 4B). These findings all demonstrate that patients in Cluster2 are associated with poor prognosis.

The overview of immune difference in TME is also shown in the heat map of Figure 4B. Details are displayed in Figures 4C–G. We find that immune, stromal scores and tumor purity are remarkably different among DEAS-based clusters (Figures 4C, D). It is noteworthy that Cluster2 gives higher immune and stromal scores, and lower tumor purity than Cluster0 and Cluster1. Figure 4E illustrates that density curve of immune scores is distributed at the right side of stromal scores, so immune scores are higher than stromal scores, which indicates that immune infiltration may play a predominant role in PTC tumor microenvironment (TME). Furthermore, immune cell infiltration analysis in Figure 4F exposes remarkable differences of the proportions of different immune cells across different clusters. Compared with Cluster0 and Cluster1, Cluster2 has significantly higher proportions of T Cells CD4 memory activated, T Cells regulatory (Treg), dendritic cells resting and dendritic cells activated, but much lower proportions of macrophages M2, mast cells activated and eosinophils.

Immune checkpoint molecules are enable to inhibit the function of the immune cells to promote immune escape and tumor formation (Liao et al., 2021). Immune checkpoint blockade (ICB) could remove inhibitory signals of T-cell activation and make tumor-reactive T Cells to mount an effective anti-tumor response and ICB therapies have been approved for treatment of a series of tumor types (Wei et al., 2018). In our study, we evaluated the expressions of immune checkpoints in three clusters. The result in Figure 4G shows that all of immune checkpoints are significantly up-regulated in Cluster2 compared to those in Cluster0 and Cluster1. Figure 4H shows that the TMB of patients belonging to Cluster2 is prominently higher than those belonging to Cluster0 and Cluster1. Then, subclass mapping method (SubMap) analysis in Figure 4I manifests that patients in Cluster2 share a higher similarity with the expression profile of patients that are responsive to PD-1 inhibitor treatment (*p*-value = 0.035). These findings validate that PTC patients in Cluster2 might be more likely to respond to anti-PD1 therapy than patients in Cluster0 and Cluster1.

**FIGURE 5:**
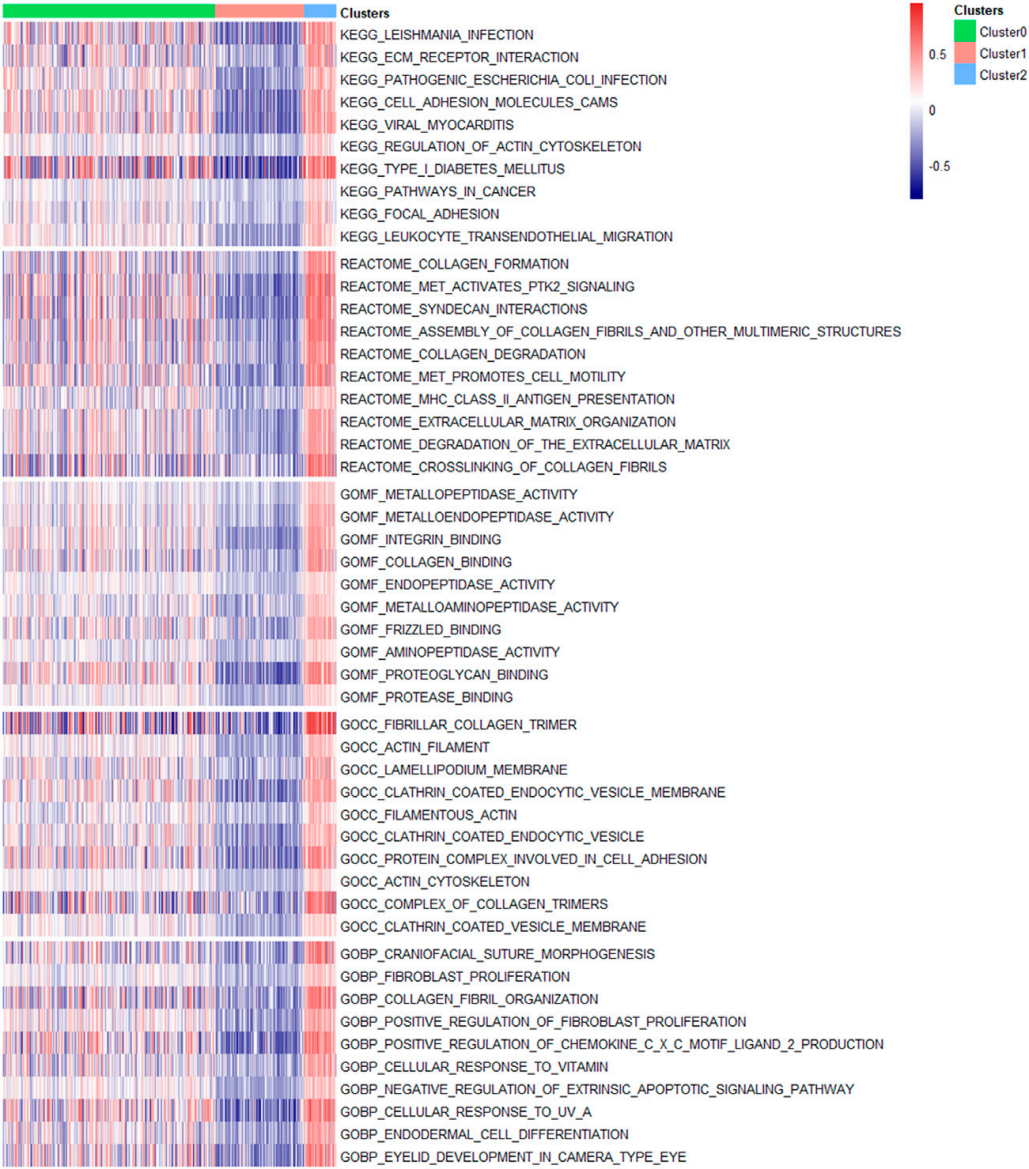
Molecular signatures associated with three DEAS-based clusters. Top 10 significantly differential signatures including KEGG pathways, reactome, molecular function, cellular component and biological process GO terms of three clusters were visualized in a matrix heatmap. The color (blue to red) in the matrix heatmap represents GSVA scores of biological pathways and GO terms of each PTC patient.

Finally, we conducted GSVA and difference analysis to screen the significantly differential pathways and biological functions across the three new subclasses. Figure 5 shows a set of cancer-related and immune-associated signatures that are different between DEAS-based clusters. For KEGG pathways, compared with Cluster0 and Cluster1, GSVA reveals that Cluster2 contains a prominent activation in cancer-related signaling pathways, including cell adhesion molecules, ECM receptor interaction, focal adhesion, pathways in cancer. We then validated the differential KEGG pathways among three clusters by conducting gene set enrichment analysis (GSEA), and the results indicate that cancer-associated pathways are significantly enriched in Cluster2 (Supplementary File S1; Supplementary Figure S3), such as cell adhesion molecules, ECM receptor interaction and focal adhesion. Except the cancer hallmark biological processes, Cluster2 is also involved in various immune-associated pathways, such as regulation of actin cytoskeleton and leukocyte transendothelial migration. For reactome, MET activates PTK2 signaling, MET promotes cell motility, MHC class II antigen presentation and extracellular matrix organization are significantly enriched in Cluster2, so it may be more likely to metastasize compared to Cluster0 and Cluster1. Besides, significant molecular function terms in Cluster2 include metallopeptidase activity, intergrin binding and Frizzled binding and significant cellular component terms are lamellpodium membrane, protein complex involved in cell adhesion and actin cytoskeleton. The molecular function analysis suggests that Cluster2 shows significant differences of biological functions from other two clusters and it may be associated with tumor growth and metastasis.

**FIGURE 6:**
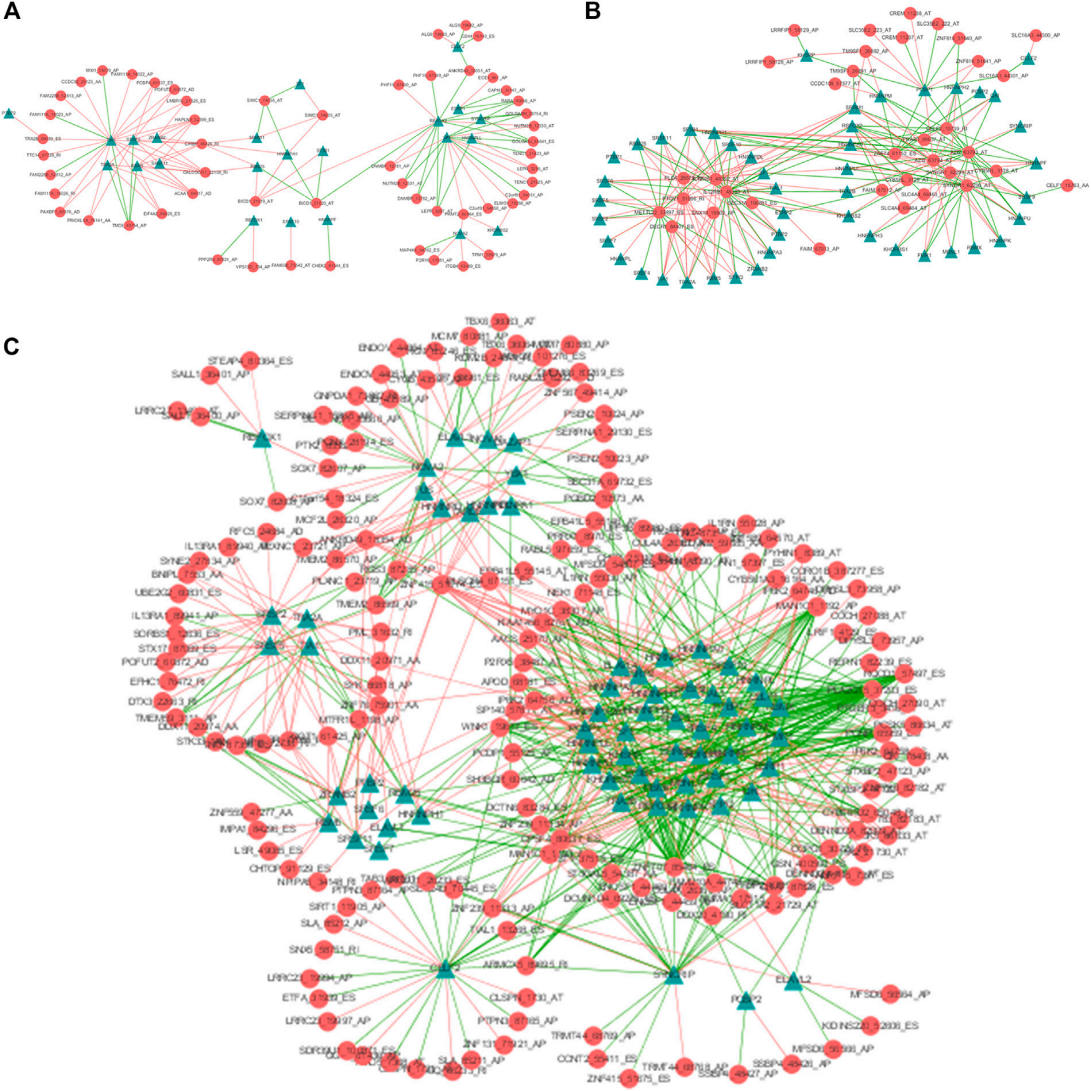
The AS-SF regulatory networks of Cluster0 **(A)**, Cluster1 **(B)** and Cluster2 **(C)**. Red circles are AS events associated with survival times and Green triangles are SFs related with corresponding AS events. The red/green lines represent positive/negative correlations between PSI values of prognostic AS events and expressions of SFs.

SFs could bind to pre-mRNAs and regulate RNA splicing *via* influencing exon selection and splicing sites. In order to portray the potential regulatory network between AS events and SFs, univariate Cox regression analysis was carried out to identify the survival-associated AS events in different clusters. Significant correlations with |R| > 0.4 and *p*-value <0.05 are shown in the network map (Figures 6A–C). A total of 55 AS events are significantly correlated with 23 SFs in Cluster0, 35 AS events with 46 SFs in Cluster1 and 175 AS events with 66 SFs in Cluster2. Notably, we can see that the majority of SFs are prominently linked with multiple AS events and a single AS events could be regulated by many different SFs. Moreover, it is visual that the relationships between SFs and AS events in Cluster2 are more complicated than those in Cluster0 and Cluster1. By network degree analysis, the average degree of nodes in Culter2 network is 5.2 that is much higher than those in Cluster0 and Cluster1, further validating that there may be more complicated regulation relationships between AS and SFs in Cluster2.

### 3.6 Prognostic value of DEAS events and establishment of prognostic model

**FIGURE 7:**
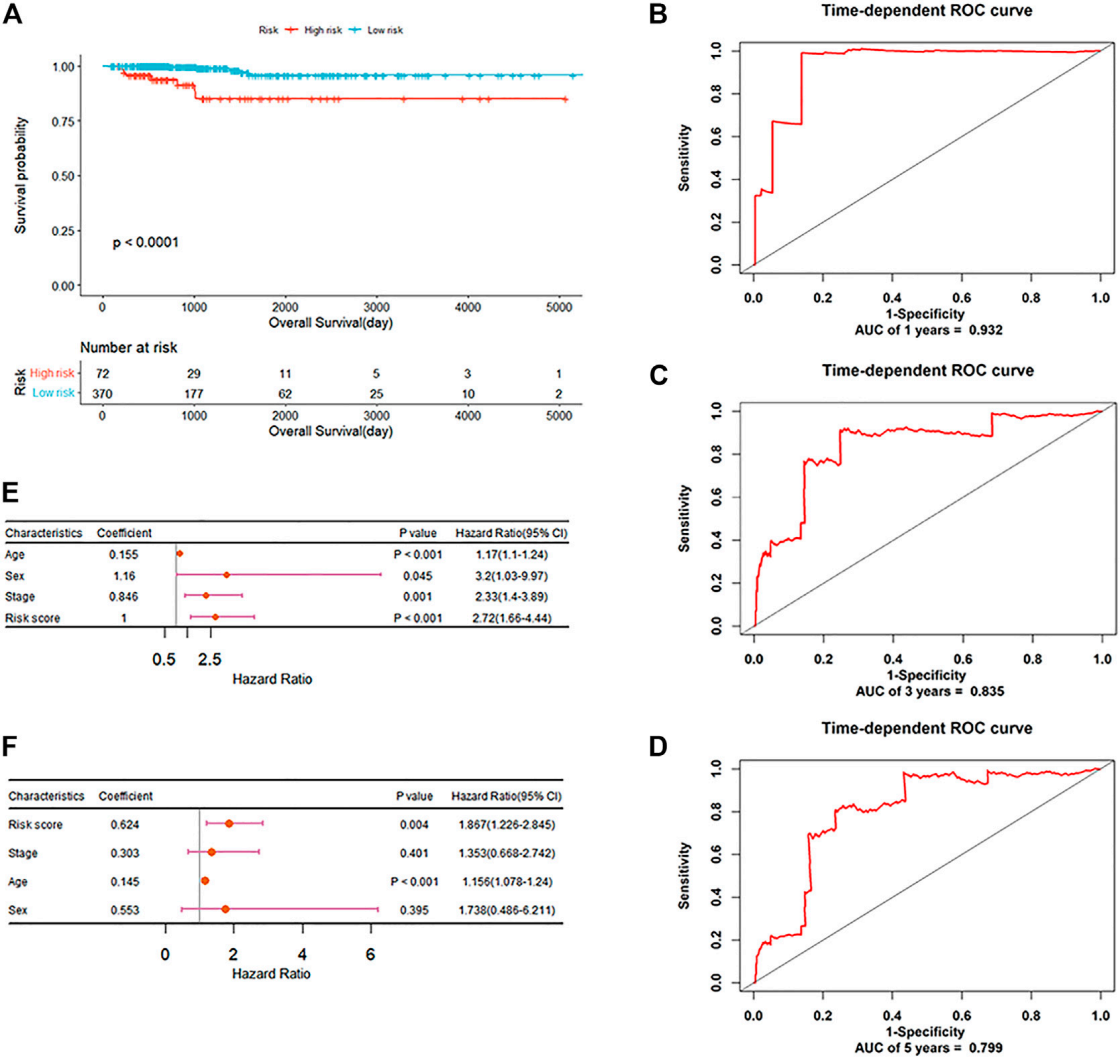
The prognostic significance of DEAS events **(A)** Kaplan-Meier curve analysis for OS **(B–D)** ROC validation of the predictive signature for predicting outcomes of PTC at 1-year, 3-year and 5-year, respectively **(E)** Forest plot summary of univariable analysis of sex, age, stage and risk score **(F)** Forest plot summary of multivariable analysis of stage and risk score.

In order to explore the potential prognostic values of the six DEAS events, the underlying relationships were investigated between DEAS events and OS of PTC by depicting Kaplan-Meier curves. Supplementary File S1; Supplementary Figures S4A–F show that three DEAS events including KIAA1217_10995_AP, DCN_23655_AT and RCAN2_76415_AP are prominently associated with OS. Then, stepwise multivariate Cox regression analysis was implemented and a predictive signature consisted of KIAA1217_10995_AP and RCAN2_76415_AP was obtained. The risk score of each patient was calculated and all patients were divided into high and low-risk groups using the cutoff value determined by X-tile. It can be seen from Figure 7A that patients with high-risk score have a lower survival rate compared with those in low-risk group (*p*-value<0.0001). For the clinicophthogical feature analysis, we have demonstrated that patients in Cluster2 shows poor prognosis, since most of patients belong to stage III and IV. The time-dependent receiver operating characteristic curve (ROC) in Figures 7B–D indicates the area under curve (AUC) values of this prognostic model in predicting outcomes at 1-year, 3-year and 5-year are 0.932, 0.835 and 0.799 respectively. The C-index of this signature reaches to as high as 0.842. Besides, the stratification analysis in Figures 7E, F was conducted based on risk score, age, sex and TNM stage. After multivariate adjustment by clinical factors, the prognostic signature could be a moderate and independent prognostic indicator for PTC patient survival. We can see that the six DEAS events could not only be the biomarkers for subclassification, but also can be considered as the prognostic predictors for PTC.

### 3.7 Determination of therapeutic drugs by CMap analysis

**FIGURE 8:**
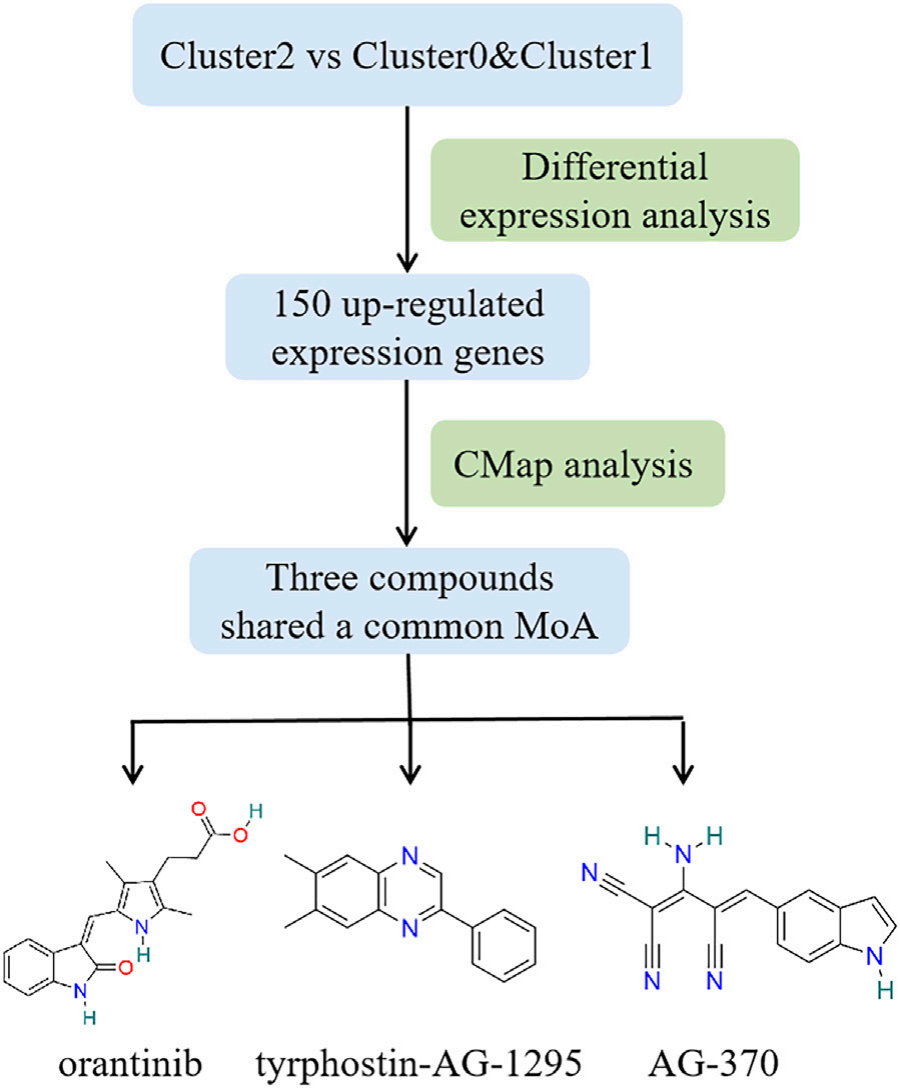
Workflow of selecting potential compounds for PTC therapy and structures of three bioactive chemicals that share common PDGFR receptor inhibitor.

Based on various analysis, we find that patients belonging to Cluster2 show the worse survival rate, more advanced clinicopathologic characteristics and more cancer hallmark pathways than those of Cluster0 and Cluster1. Discovering new effective drugs for patients in Cluster2 could be of more practical significance and further improve the prognosis of PTC. Here, a flow chart is given in Figure 8 showing the selection process of potential compounds for PTC clinical therapy. Firstly, differential gene expression analysis was performed and the top 150 up-regulated expression genes were screened in Cluster2 compared to Cluster0 and Cluster1. Those were predicted as the potential drugs that can reverse the expression of these genes by CMap analysis. It is expected that these predicted bioactive chemicals might be of great potential in the therapy of patients in Cluster2. A negative connectivity score indicates that the compound represses the query gene expression. The prediction result demonstrates that there are 45 different modes of action (MoA) in top 50 compounds with the lowest scores. Furthermore, three compounds including orantinib, tyrphostin-AG-1295 and AG-370 with connectivity scores close to -1 share a common action mode of PDGFR receptor inhibitor and targeted a common gene, PDGFRB. The detailed information about them is listed in Supplementary File S1; Supplementary Table S4.

## 4 Discussion

As the main contributor to overall thyroid cancer, PTC is the only histological subtype that greatly increase in all countries (Miranda-Filho et al., 2021). Besides, PTC is a heterogeneous neoplasm both at histological and molecular levels. Different PTC subtypes exhibit various clinical manifestation and prognosis. Therefore, it is of great significance to find more sophisticated molecular subclassifications and to identify novel biomarkers associated with PTC subtypes for precise diagnosis, surgical and medical therapy of PTC patients. Recent researches have studied genome-wide AS landscape in cancers and AS events are proved to be involved in tumorigenesis and prognosis (Lin et al., 2019; Han et al., 2021; Zheng et al., 2021). Guo et al. have firstly integrated gene expression and transcriptome AS profiles to identify breast cancer subtypes (Guo et al., 2019). Zhao et al. divided glioblastoma into two subtypes based on the prognostic AS events by combining similarity network fusion and consensus clustering (Zhao et al., 2021). Jun et al. extracted the DEAS events between gastric tumors and matched normal mucosa, and classified gastric cancers into three subtypes (Jun et al., 2021). For PTC, Zheng et al. identified cancer-associated AS events by comparing AS events’ PSI values between tumors and adjacent normal tissues (Zheng et al., 2021). Recently, Park et al. have discovered two AS events which could be potential biomarkers for PTC subtypes classification, which indicates that AS events may be additional classifiers except the histological or molecular subtypes (Park et al., 2022). However, the deeper insights into AS events and their potential of cancer classification biomarkers for PTC subtypes remain to be further explored. In this study, we present a new molecular subclassification and then performed *in silico* predictions for precise diagnosis and prognosis of PTC based on AS profiles.

Firstly, we identified six DEAS events among three histological PTC variants. Among them, it has been confirmed that TUBB3_38175_ES could be a potential biomarker for PTC subclassification and characterization (Park et al., 2022). Coding for a microtubule protein, TUBB3 is overexpressed and related to poor prognoses in various cancers (Li et al., 2021). Zhang et al. suggest that exon skip in DCN is associated with patient survival with glioblastoma (Zhang et al., 2021a). KIAA1217 shows different splicing in esophageal squamous cell carcinoma (Ding et al., 2021) and intron retention of KIAA1217 has been found in non-small cell lung cancer, but it is not supported by results of RT-PCR (Langer et al., 2010). These findings reveal the potential values of DEAS events with molecular typing and prognosis in various cancers. However, both PCA analysis and the *in silico* prediction model show that three variants cannot be well partitioned by the six DEAS events. Furthermore, due to the molecular heterogeneity, the multiple growth pattern cell types and stromal changes of PTC, the current histological subtypes still have some limitations. So we attempt to present a new subclassification for PTC based on the six DEAS events and k-means clustering analysis indicates that based on the six DEAS events, all PTC patient samples can be clearly clustered into new three groups, named as Cluster0, Cluster1 and Cluster2 respectively. We compared our new clusters with current histological variants and the previously proposed four DNA promoter methylation-based classes and found that they are new subtypes different from the existing variants. To validate the classification performance of DEAS events for the new PTC groups, a SVM classifier was constructed and it yields a promising performance for distinguishing the three groups with the overall accuracy of 91.2%.

Next, we need to prove the validity of the proposed new subcalssification for PTC. So a comprehensive difference analysis was performed in terms of clinical, immune and molecular features among three groups. Overall, Cluster2 yields significant differences from Cluster0 and Cluster1. The clinicopathology analysis shows that patients in Cluster2 have the worse OS and most of them belong to the advanced stages of III and IV. So Cluster2 is associated with poor prognosis of PTC, which is also indicated by the immune characteristics that Cluster2 with poor prognosis gives higher immune and stromal scores and lower tumor purity. Previous reports indicat that lower tumor purity tends to yield worse survival in glioma and colon cancer (Zhang et al., 2017; Mao et al., 2018). Mao et al. also have observed that PTC patients with poor OS show higher immune and stromal scores, while lower tumor purity, which is consistent with our result (Mao et al., 2021). Besides, stromal cell-derived factor 1 involved in tumor cell migration and metastasis and might promote tumorogenesis, invasion and metastasis of PTC (Liu et al., 2012). However, it has been indicated that stromal secreted SOD3 could stimulate cancer cell growth and inhibit cancer cell migration (Parascandolo et al., 2017), and high stromal score was significantly associated with improved PFS in patients with PTC (Tang et al., 2021). So the effect of stromal cells in PTC remains to be further elucidated. We demonstrated that immune infiltration may play a predominant role in PTC TME and previous researches have reported that immune cells, such as Tregs, dendritic cells and T Cells CD4 memory activated could promote tumor development and metastasis (Yu et al., 2013; Zhang et al., 2020; Li et al., 2022). Tregs can facilitate tumor cells to escape immune surveillance by being recruited to the tumor microenvironment and inhibiting Tregs function has been a viable strategy to enhance antitumor immunity (Dees et al., 2021). The study of Zhang et al. (Zhang et al., 2020) demonstrates that the dendritic cells resting and dendritic cells activated are observed to be associated with poor prognosis in thyroid cancer. Meanwhile, we know that TMB reflects cancer mutation quantity and it has been proposed that higher TMB clinically correlates with better immune checkpoint inhibitor (ICI) outcomes because higher TMB results in more neo-antigens and increases chances for T Cell recognition (Yarchoan et al., 2019; Jardim et al., 2021). The analysis on immune checkpoint expressions and TMB disclosed that Cluster2 would probably benefit more from immune checkpoints blockade, and patients in Cluster2 are proved to be more responsive to PD-1 inhibitor treatment by SubMap analysis. Finally, a set of cancer-associated and immune-related pathways and biological functions were enriched in Cluster2, such as cell adhesion molecules, focal adhesion, MET activates PTK2 signaling and leukocyte transendothelial migration, indicating that patients in Cluster2 may be more likely to metastasis and immune response.

It has been shown that somatic mutations and overexpression of SFs contribute to tumorigenesis (Lee and Rio, 2015; Sveen et al., 2016), so they play a vital role in the malignant transformation of cancer through modulating the oncogenic variants (Xie et al., 2021). To inquire the mechanisms of the effects of SFs on alternative splicing, we investigated the relationships between survival-associated AS and SFs *via* constructing splicing correlation networks for the three groups. It suggests that a single SF could regulate different AS events and the same AS events could be controlled by various SFs. Moreover, almost all SFs were correlated with survival-related AS events in Cluster2, which validates that there might be more complicated regulation relationships between AS and SFs in Cluster2.

No significant OS difference has been manifested among the current histological variants of CPTC, FPTC and TCPTC by Kaplan-Meier analysis (Supplementary File S1; Supplementary Figure S5). However, the new groups classified by the six DEAS events exhibits significant OS differences (Figure 4A). So we would like to verify the prognostic values of the six DEAS events. Three DEAS events were found to be significantly correlated with OS. KIAA1217_10995_AP and RCAN2_76415_AP are associated with poor prognosis and DCN_23655_AT with good prognosis. So, a prognostic signature was established for effective prediction of PTC’s OS. The Kaplan-Meier analysis suggests that high-risk patients had a shorter survival times than low-risk patients. Furthermore, the ROC curves and C-index demonstrate powerful predictive ability of the signature with AUC of 0.932, 0.835 and 0.799 for 1, 3 and 5 years respectively. Additionally, the risk score is an independent applicable prognostic indicator of PTC after adjusting for clinical factors including age, sex and stage. Overall, we can conclude that the six DEAS events identified by us could be the potential biomarkers for PTC subclassification and characterization as well as the prognostic predictors of PTC patients.

Finally, due to the worse clinical course of patients belonging to Cluster2, CMap analysis was performed to disclose the potential drugs that might reverse the expression of genes which are highly expressed in Cluster2, so as to improve the prognosis of patients in Cluster2. Three compounds (orantinib, tyrphostin-AG-1295, and AG-370) were identified and all of them are receptor tyrosine kinase inhibitors. In order to support the potential of them in the practical therapy of PTC and other cancers, a literature-searching was conducted. Among three compounds, orantinib is a small-molecule, orally administered, multiple-receptor tyrosine kinase inhibitor of PDGF receptor-β and VEGF receptor-2 (Kudo et al., 2018). Previous studies have shown that orantinib may influence tumor growth by multiple mechanisms including inhibition of endothelial cell proliferation and/or survival as well as tumor cell and stromal cell proliferation and had a therapeutic effect on a variety of solid tumors and hematological malignancies (Laird et al., 2000; Wang et al., 2013). Tyrphostin-AG-1295 is also an specific inhibitor of PDGF receptor pathways and could reduce neointimal formation in aortic allograft vasculopathy by inhibiting PDGF-β-triggered tyrosine phosphorylation (Karck et al., 2002). AG-370, an inhibitor of PDGF receptor kinase activity, could repress PDGF-induced and 17beta-retradiol-induced gonocyte proliferation (Thuillier et al., 2010). Overall, all three compounds were receptor tyrosine kinase inhibitors, so they were predicted to target the common gene of PDGFRB in our analysis. PDGFRB is a member of receptor protein tyrosine kinase (RPTKs) III family and stimulation of the PDGFR could lead to activation of intracellular signaling pathways that can promote cell migration, invasion, survival and proliferation (Steller et al., 2013). Besides, PDGFRB plays a pivotal role in angiogenesis and tumor cell proliferation. It has been proposed that PDGF-mediated angiogenesis appears to be a reasonable target in the treatment of PTCs (Durante et al., 2011; Liu et al., 2021). All could prove the validity of our prediction results, but the practical applicability of those compounds on PTC still need to explore by experiment in future studies.

Overall, this study provides a deeper understanding of the potential value of AS events for PTC characterization, which might contribute to our cognition about the potential mechanism of AS events in the development of PTC. However, the potential clinical diagnostic and prognostic value of these biomarkers for PTC still need to be validated based on clinical trial or biological experiments. For all this, our research presents a novel perspective for PTC subclassification and identified potential therapeutic compounds for PTC.

## 5 Conclusion

We proposed a new molecular subclassification including three subtypes for PTC based on AS profiles and identified six DEAS events as the potential biomarkers for precise diagnosis and probable prognostic predictors of PTC. In addition, a DEAS-based classifier was constructed for distinguishing the three new clustering groups using SVM, which yields a promising performance. A series of analyses indicate that there are significant differences of clinicopathology, molecular and immune characteristics among the new groups. And samples in Cluster2 showed poor prognosis, higher immune heterogeneity and more sensitive for anti-PD1 therapy. Finally, three potential bioactive chemicals were screened out, including orantinib, tyrphostin-AG-1295, and AG-370, which might provide potential treatment of PTC patients. As a whole, this research increases the understanding of the effect of AS events on PTC characterization, as well as provides new insights for refining precise subclassification and improving medical therapy for PTC patients.

## Data Availability

The original contributions presented in the study are included in the article/Supplementary Material, further inquiries can be directed to the corresponding author.

## Glossary

AA: alternate acceptor site
ACC: accuracy
AD: alternate donor site
AP: alternate promoter;
AS: alternative splicing
AT: alternate terminator
AUC: area under curve
C-index: concordance index
CMap: connectivity map
CPTC: classical papillary thyroid cancer
DEAS: differentially expressed alternative splicing
ES: exon skip
FN: false negative
FP: false positive
FPTC: follicular papillary thyroid cancer
GO: gene ontology
GSEA: Gene set enrichment analysis
GSVA: Gene set variation analysis
ICB: immune checkpoint blockade
ICI: immune checkpoint inhibitor
KEGG: Kyoto Encylopaedia of Genes and Genomes
MoA: modes of action
ME: mutually exclusive exons
MSigDB: molecular signatures database
OS: overall survival
PCA: principle component analysis
PTC: papillary thyroid cancer
ROC: receiver operating characteristic
PSI: percent-spliced-in
RI: retained intron
SFs: splicing factors
SS: survival status
SubMap: subclass mapping method
SVM: support vector machine
TCPTC: tall cell papillary thyroid cancer
TP: true positive
TN: true negative
TMB: tumor mutational burden

## References

[B1] Agrawal N. Akbani R. Aksoy B. A. Ally A. Arachchi H. Asa, Sylvia L. (2014). Integrated genomic characterization of papillary thyroid carcinoma. Cell 159, 676–690. 10.1016/j.cell.2014.09.050 25417114 PMC4243044

[B2] Bonnal S. C. López-Oreja I. Valcárcel J. (2020). Roles and mechanisms of alternative splicing in cancer — Implications for care. Nat. Rev. Clin. Oncol. 17, 457–474. 10.1038/s41571-020-0350-x 32303702

[B3] Dees S. Ganesan R. Singh S. Grewal I. S. (2021). Regulatory T cell targeting in cancer: Emerging strategies in immunotherapy. Eur. J. Immunol. 51, 280–291. 10.1002/eji.202048992 33302322

[B4] Ding J. Li C. Cheng Y. Du Z. Wang Q. Tang Z. (2021). Alterations of RNA splicing patterns in esophagus squamous cell carcinoma. Cell & Biosci. 11, 36. 10.1186/s13578-021-00546-z PMC7871539 33563334

[B5] Durante C. Tallini G. Puxeddu E. Sponziello M. Moretti S. Ligorio C. (2011). BRAFV600E mutation and expression of proangiogenic molecular markers in papillary thyroid carcinomas. Eur. J. Endocrinol. 165, 455–463. 10.1530/EJE-11-0283 21653734

[B6] Fagin J. A. Wells S. A. (2016). Biologic and clinical perspectives on thyroid cancer. N. Engl. J. Med. 375, 1054–1067. 10.1056/NEJMra1501993 27626519 PMC5512163

[B7] Guo Y. Shang X. Li Z. (2019). Identification of cancer subtypes by integrating multiple types of transcriptomics data with deep learning in breast cancer. Neurocomputing 324, 20–30. 10.1016/j.neucom.2018.03.072

[B8] Han B. Yang M. Yang X. Liu M. Xie Q. Fan G. (2021). Systematic analysis of survival-associated alternative splicing signatures in thyroid carcinoma. Front. Oncol. 11, 561457. 10.3389/fonc.2021.561457 34249669 PMC8261059

[B9] Hänzelmann S. Castelo R. Guinney J. (2013). Gsva: Gene set variation analysis for microarray and RNA-seq data. BMC Bioinforma. 14, 7. 10.1186/1471-2105-14-7 PMC3618321 23323831

[B10] Hoshida Y. Brunet J. P. Tamayo P. Golub T. R. Mesirov J. P. (2007). Subclass mapping: Identifying common subtypes in independent disease data sets. PLoS One 2, e1195. 10.1371/journal.pone.0001195 18030330 PMC2065909

[B11] Jardim D. L. Goodman A. De Melo Gagliato D. Kurzrock R. (2021). The challenges of tumor mutational burden as an immunotherapy biomarker. Cancer Cell 39, 154–173. 10.1016/j.ccell.2020.10.001 33125859 PMC7878292

[B12] Jun Y. Suh Y.-S. Park S. Lee J. Kim J.-I. Lee S. (2021). Comprehensive analysis of alternative splicing in gastric cancer identifies epithelial–mesenchymal transition subtypes associated with survival. Cancer Res. 82, 543–555. 10.1158/0008-5472.CAN-21-2117 PMC9359730 34903603

[B13] Karck M. Meliss R. Hestermann M. Mengel M. Pethig K. Levitzki A. (2002). Inhibition of aortic allograft vasculopathy by local delivery of platelet-derived growth factor receptor tyrosine-kinase blocker AG-1295. Transplantation 74, 1335–1341. 10.1097/00007890-200211150-00023 12451275

[B14] Kudo M. Cheng A.-L. Park J.-W. Park J. H. Liang P.-C. Hidaka H. (2018). Orantinib versus placebo combined with transcatheter arterial chemoembolisation in patients with unresectable hepatocellular carcinoma (ORIENTAL): A randomised, double-blind, placebo-controlled, multicentre, phase 3 study. Lancet Gastroenterology Hepatology 3, 37–46. 10.1016/S2468-1253(17)30290-X 28988687

[B15] Laird A. D. Vajkoczy P. Shawver L. K. Thurnher A. Liang C. Mohammadi M. (2000). SU6668 is a potent antiangiogenic and antitumor agent that induces regression of established tumors. Cancer Res. 60, 4152–4160. 10945623

[B16] Langer W. Sohler F. Leder G. Beckmann G. Seidel H. Gröne J. (2010). Exon Array Analysis using re-defined probe sets results in reliable identification of alternatively spliced genes in non-small cell lung cancer. BMC Genomics 11, 676. 10.1186/1471-2164-11-676 21118496 PMC3053589

[B17] Lee Y. Rio D. C. (2015). Mechanisms and regulation of alternative pre-mRNA splicing. Annu. Rev. Biochem. 84, 291–323. 10.1146/annurev-biochem-060614-034316 25784052 PMC4526142

[B18] Li H. Yang J. Yang G. Ren J. Meng Y. Qi P. (2021). Identification of prognostic alternative splicing events in sarcoma. Sci. Rep. 11, 14949. 10.1038/s41598-021-94485-x 34294833 PMC8298452

[B19] Li M. Zhao J. Yang R. Cai R. Liu X. Xie J. (2022). CENPF as an independent prognostic and metastasis biomarker corresponding to CD4+ memory T cells in cutaneous melanoma. Cancer Sci. 113, 1220–1234. 10.1111/cas.15303 35189004 PMC8990861

[B20] Liao G. Wang P. Wang Y. (2021). Identification of the prognosis value and potential mechanism of immune checkpoints in renal clear cell carcinoma microenvironment. Front. Oncol. 11, 720125. 10.3389/fonc.2021.720125 34336706 PMC8317210

[B21] Lin P. He R.-Q. Huang Z.-G. Zhang R. Wu H.-Y. Shi L. (2019). Role of global aberrant alternative splicing events in papillary thyroid cancer prognosis. Aging 11, 2082–2097. 10.18632/aging.101902 30986203 PMC6503875

[B22] Liu B. Xiao X. Lin Z. Lou Y. Zhao L. (2021). PDGFRB is a potential prognostic biomarker and correlated with immune infiltrates in gastric cancer. Cancer Biomarkers 34, 251–264. Preprint. 10.3233/CBM-210335 PMC12364286 34958001

[B23] Liu Z. Rabadan R. (2021). Computing the role of alternative splicing in cancer. Trends Cancer 7, 347–358. 10.1016/j.trecan.2020.12.015 33500226 PMC7969404

[B24] Liu Z. Sun D.-X. Teng X.-Y. Xu W.-X. Meng X.-P. Wang B.-S. (2012). Expression of stromal cell-derived factor 1 and CXCR7 in papillary thyroid carcinoma. Endocr. Pathol. 23, 247–253. 10.1007/s12022-012-9223-x 23070788

[B25] Lloyd R. V. Buehler D. Khanafshar E. (2011). Papillary thyroid carcinoma variants. Head Neck Pathology 5, 51–56. 10.1007/s12105-010-0236-9 21221869 PMC3037461

[B26] Mao M. Huang R.-Z. Zheng J. Liang H.-Q. Huang W.-H. Liu J. (2021). OGDHL closely associates with tumor microenvironment and can serve as a prognostic biomarker for papillary thyroid cancer. Cancer Med. 10, 728–736. 10.1002/cam4.3640 33405394 PMC7877349

[B27] Mao Y. Feng Q. Zheng P. Yang L. Liu T. Xu Y. (2018). Low tumor purity is associated with poor prognosis, heavy mutation burden, and intense immune phenotype in colon cancer. Cancer Manag. Res. 10, 3569–3577. 10.2147/CMAR.S171855 30271205 PMC6149864

[B28] Mayakonda A. Lin D. C. Assenov Y. Plass C. Koeffler H. P. (2018). Maftools: Efficient and comprehensive analysis of somatic variants in cancer. Genome Res. 28, 1747–1756. 10.1101/gr.239244.118 30341162 PMC6211645

[B29] Miranda-Filho A. Lortet-Tieulent J. Bray F. Cao B. Franceschi S. Vaccarella S. (2021). Thyroid cancer incidence trends by histology in 25 countries: A population-based study. Lancet Diabetes & Endocrinol. 9, 225–234. 10.1016/S2213-8587(21)00027-9 33662333

[B30] Parascandolo A. Rappa F. Cappello F. Kim J. Cantu D. A. Chen H. (2017). Extracellular superoxide dismutase expression in papillary thyroid cancer mesenchymal stem/stromal cells modulates cancer cell growth and migration. Sci. Rep. 7, 41416. 10.1038/srep41416 28216675 PMC5316948

[B31] Park J. Kim D. Lee J.-O. Park H.-C. Ryu B. Y. Kim J. H. (2022). Dissection of molecular and histological subtypes of papillary thyroid cancer using alternative splicing profiles. Exp. Mol. Med. 54, 263–272. 10.1038/s12276-022-00740-0 35277656 PMC8980103

[B32] Prat A. Navarro A. Paré L. Reguart N. Galván P. Pascual T. (2017). Immune-related gene expression profiling after PD-1 blockade in non–small cell lung carcinoma, head and neck squamous cell carcinoma, and melanoma. Cancer Res. 77, 3540–3550. 10.1158/0008-5472.CAN-16-3556 28487385

[B33] Pusztaszeri M. Auger M. (2017). Update on the cytologic features of papillary thyroid carcinoma variants. Diagn. Cytopathol. 45, 714–730. 10.1002/dc.23703 28262004

[B34] Ritchie M. E. Phipson B. Wu D. Hu Y. Law C. W. Shi W. (2015). Limma powers differential expression analyses for RNA-sequencing and microarray studies. Nucleic Acids Res. 43, e47. 10.1093/nar/gkv007 25605792 PMC4402510

[B35] Sciarrillo R. Wojtuszkiewicz A. Assaraf Y. G. Jansen G. Kaspers G. J. L. Giovannetti E. (2020). The role of alternative splicing in cancer: From oncogenesis to drug resistance. Drug Resist. Updat. 53, 100728. 10.1016/j.drup.2020.100728 33070093

[B36] Shi X. Liu R. Basolo F. Giannini R. Shen X. Teng D. (2016). Differential clinicopathological risk and prognosis of major papillary thyroid cancer variants. J. Clin. Endocrinol. Metabolism 101, 264–274. 10.1210/jc.2015-2917 PMC4701842 26529630

[B37] Steller E. J. A. Raats D. A. Koster J. Rutten B. Govaert K. M. Emmink B. L. (2013). PDGFRB promotes liver metastasis formation of mesenchymal-like colorectal tumor cells. Neoplasia 15, 204–217. 10.1593/neo.121726 23441134 PMC3579322

[B38] Sveen A. Kilpinen S. Ruusulehto A. Lothe R. A. Skotheim R. I. Ruusulehto A. (2016). Aberrant RNA splicing in cancer; expression changes and driver mutations of splicing factor genes. Oncogene 35, 2413–2427. 10.1038/onc.2015.318 26300000

[B39] Tallini G. Tuttle R. M. Ghossein R. A. (2016). The history of the follicular variant of papillary thyroid carcinoma. J. Clin. Endocrinol. Metabolism 102, 15–22. 10.1210/jc.2016-2976 27732333

[B40] Tang J. Jiang S. Gao Q. Xi X. Gao L. Zhao R. (2021). Development and validation of a nomogram based on stromal score to predict progression-free survival of patients with papillary thyroid carcinoma. Cancer Med. 10, 5488–5498. 10.1002/cam4.4112 34240816 PMC8366082

[B41] Thuillier R. Mazer M. Manku G. Boisvert A. Wang Y. Culty M. (2010). Interdependence of platelet-derived growth factor and estrogen-signaling pathways in inducing neonatal rat testicular gonocytes proliferation. Biol. Reproduction 82, 825–836. 10.1095/biolreprod.109.081729 PMC2857630 20089883

[B42] Wang L. Liu Z. Ma D. Piao Y. Guo F. Han Y. (2013). SU6668 suppresses proliferation of triple negative breast cancer cells through down-regulating MTDH expression. Cancer Cell Int. 13, 88. 10.1186/1475-2867-13-88 23984913 PMC3844503

[B43] Wang X. Fu X. Zhang J. Xiong C. Zhang S. Lv Y. (2020). Identification and validation of m6A RNA methylation regulators with clinical prognostic value in Papillary thyroid cancer. Cancer Cell Int. 20, 203. 10.1186/s12935-020-01283-y 32514248 PMC7260751

[B44] Wei S. C. Duffy C. R. Allison J. P. (2018). Fundamental mechanisms of immune checkpoint blockade therapy. Cancer Discov. 8, 1069–1086. 10.1158/2159-8290.CD-18-0367 30115704

[B45] Xie R. Chen X. Cheng L. Huang M. Zhou Q. Zhang J. (2021). NONO inhibits lymphatic metastasis of bladder cancer via alternative splicing of SETMAR. Mol. Ther. 29, 291–307. 10.1016/j.ymthe.2020.08.018 32950106 PMC7791011

[B46] Yarchoan M. Albacker L. A. Hopkins A. C. Montesion M. Murugesan K. Vithayathil T. T. (2019). PD-L1 expression and tumor mutational burden are independent biomarkers in most cancers. JCI Insight 4, e126908. 10.1172/jci.insight.126908 30895946 PMC6482991

[B47] Yoshihara K. Shahmoradgoli M. Martínez E. Vegesna R. Kim H. Torres-Garcia W. (2013). Inferring tumour purity and stromal and immune cell admixture from expression data. Nat. Commun. 4, 2612. 10.1038/ncomms3612 24113773 PMC3826632

[B48] Yu H. Huang X. Liu X. Jin H. Zhang G. E. Zhang Q. (2013). Regulatory T cells and plasmacytoid dendritic cells contribute to the immune escape of papillary thyroid cancer coexisting with multinodular non-toxic goiter. Endocrine 44, 172–181. 10.1007/s12020-012-9853-2 23264145

[B49] Zhang B. Wu Q. Cheng S. Li W. (2021a). Systematic profiling of mRNA splicing reveals the prognostic predictor and potential therapeutic target for glioblastoma multiforme. J. Oncol. 2021, 4664955. 10.1155/2021/4664955 34326872 PMC8277521

[B50] Zhang C. Cheng W. Ren X. Wang Z. Liu X. Li G. (2017). Tumor purity as an underlying key factor in glioma. Clin. Cancer Res. 23, 6279–6291. 10.1158/1078-0432.CCR-16-2598 28754819

[B51] Zhang L. Wang Y. Li X. Wang Y. Wu K. Wu J. (2020). Identification of a recurrence signature and validation of cell infiltration level of thyroid cancer microenvironment. Front. Endocrinol. 11, 467. 10.3389/fendo.2020.00467 PMC7390823 32793117

[B52] Zhang Y. Qian J. Gu C. Yang Y. (2021b). Alternative splicing and cancer: A systematic review. Signal Transduct. Target. Ther. 6, 78. 10.1038/s41392-021-00486-7 33623018 PMC7902610

[B53] Zhao L. Zhang J. Liu Z. Wang Y. Xuan S. Zhao P. (2021). Comprehensive characterization of alternative mRNA splicing events in glioblastoma: Implications for prognosis, molecular subtypes, and immune microenvironment remodeling. Front. Oncol. 10, 555632. 10.3389/fonc.2020.555632 33575206 PMC7870873

[B54] Zheng X. Feng L. Yin Y. Yu C. He X. Zhu J. (2021). Comprehensive analysis of aberrant alternative splicing related to carcinogenesis and prognosis of papillary thyroid cancer. Aging 13, 23149–23168. 10.18632/aging.203608 34628367 PMC8544310

