# Supplementary material for: A new molecular subclassification and in silico predictions for diagnosis and prognosis of papillary thyroid cancer by alternative splicing profile
Source: Front Pharmacol. 2023 Mar 6;14:1119789. doi: 10.3389/fphar.2023.1119789 (PMC10025316; doi:10.3389/fphar.2023.1119789)
Supplement: Supplementary file 1 [file DataSheet1.docx]

**
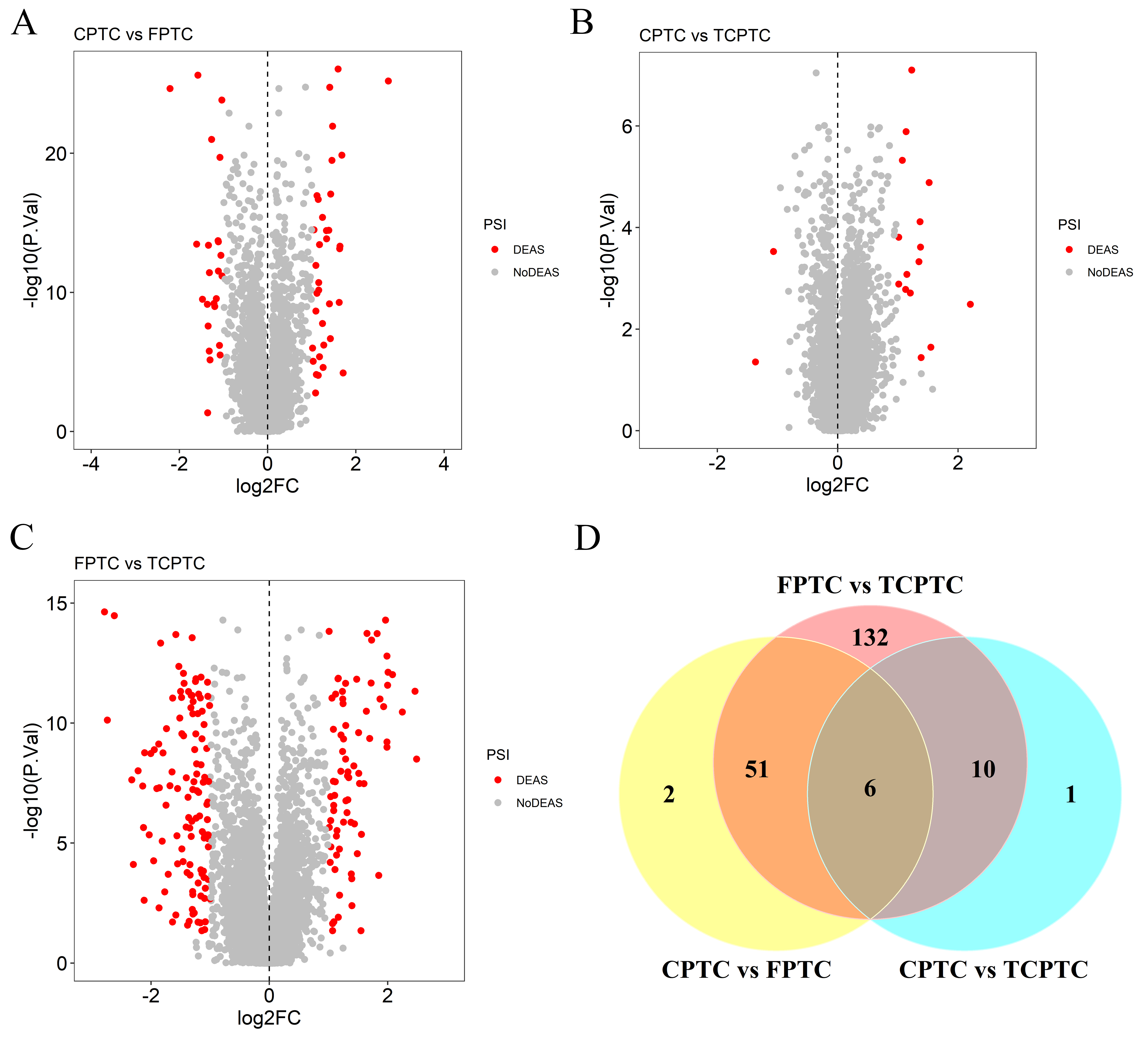
**

**Figure S1.** Selection of differentially expressed alternative splicing events among three PTC variants. **(A)** The selection of DEAS events between CPTC and FPTC. **(B)** DEAS events between CPTC and TCPTC. **(C)** DEAS events between FPTC and TCPTC. The red pots represent the DEAS events and the gray pots represent AS events with no significant difference. **(D)** The Venn diagram of DEAS events among three PTC variants.

**
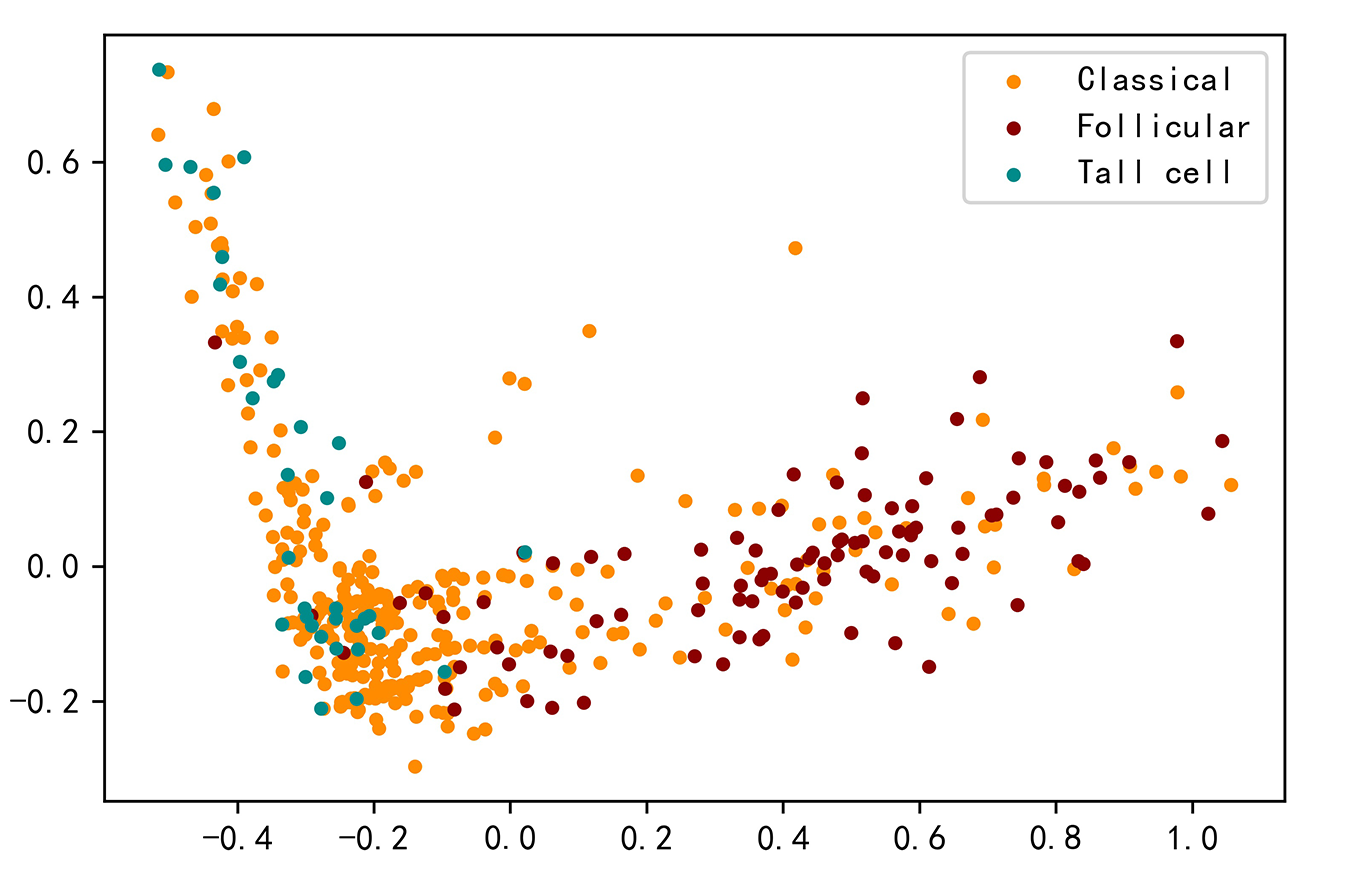
**

**Figure S2.** PCA of three PTC variants was shown in the scatter plot.

**
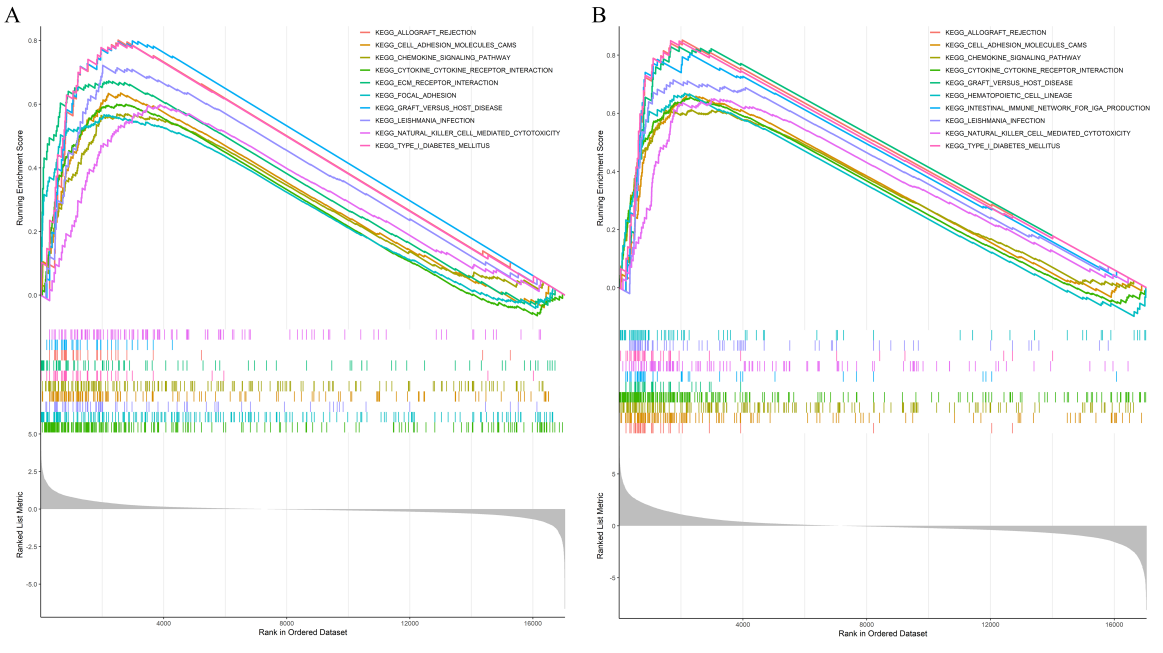
**

**Figure S3.** Top 10 significant differential KEGG pathways among three clusters by GSEA analyses. **(A)** Top 10 differential KEGG pathways between Cluster0 and Cluster2. **(B)** Top 10 differential KEGG pathways between Cluster1 and Cluster2.


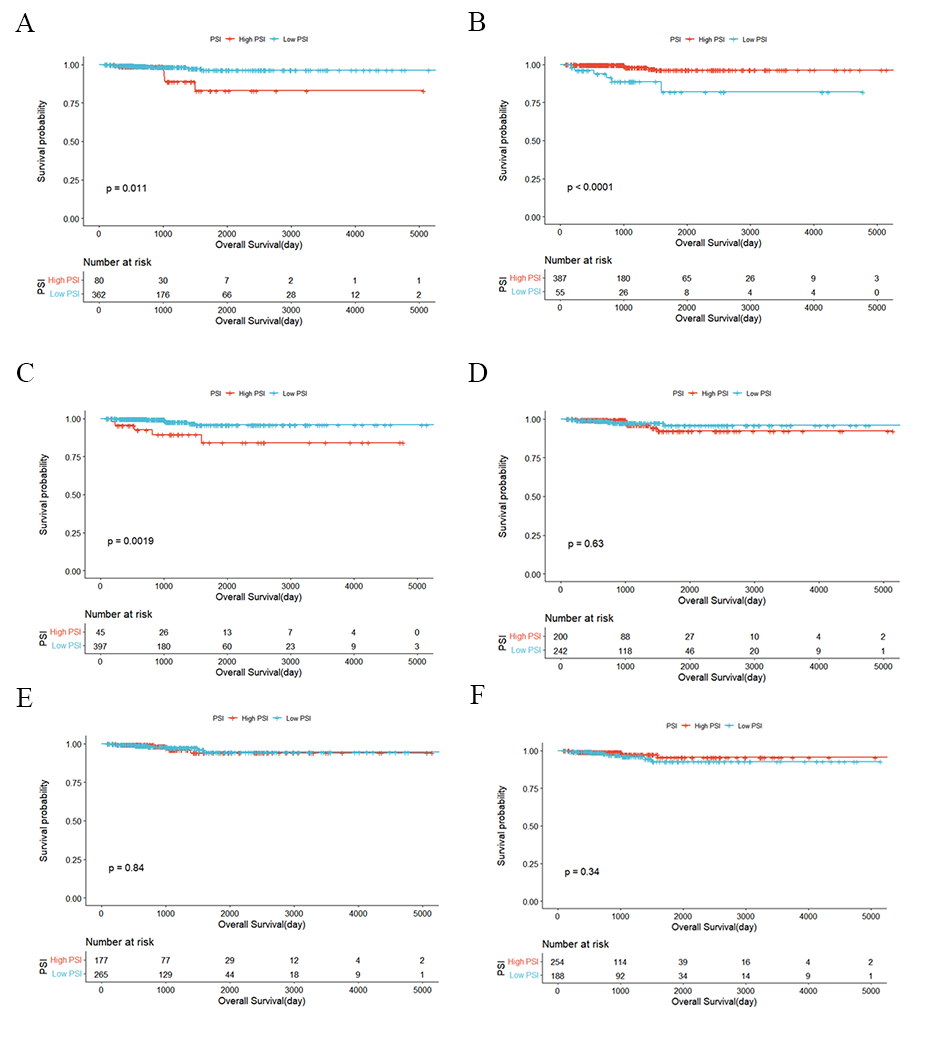


**Figure S4.** Kaplan-Meier curve analysis of six DEAS events for OS. **(A)** KIAA1217_10995_AP, **(B)** DCN_23655_AT, **(C)** RCAN2_76415_AP, **(D)** TUBB3_38175_ES, **(E)** NNMT_18817_AP, **(F)** COL14A1_85105_AP.

**
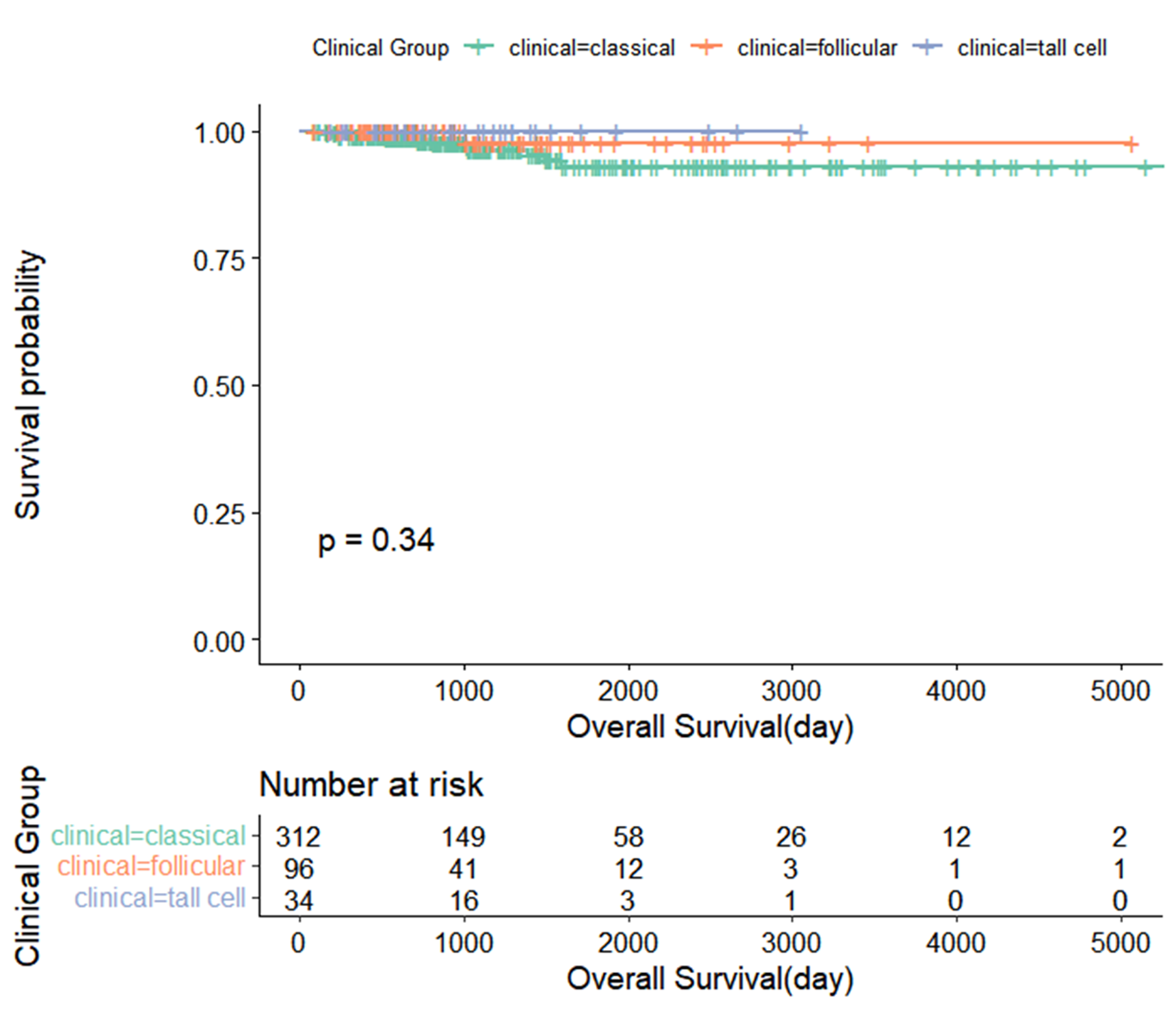
**

**Figure S5.** Kaplan-Meier survival analysis of patients within three PTC variants of OS.

**Table S1.** The splicing factors gene list collected from the SpliceAid 2.

| Gene list | SRSF7, CELF1, DAZAP1, ESRP1, ESRP2, CELF2, FMR1, RBFOX1, RBFOX2, HNRNPA0, HNRNPA1, HNRNPA2B1, HNRNPA3, HNRNPC, HNRNPC1, HNRNPC2, HNRNPD, HNRNPD0, HNRNPDL, PCBP1, PCBP2, HNRNPF, RBMX, HNRNPH1, HNRNPH2, HNRNPH3, PTBP1, HNRNPJ, HNRNPK, HNRNPL, HNRNPLL, HNRNPM, FUS, SYNCRIP, HNRNPU, TRA2A, TRA2B, ELAVL2, ELAVL3, ELAVL4, ELAVL1, KHSRP, MBNL1, NOVA1, NOVA2, PTBP2, SFPQ, QKI |
| --- | --- |

**Table S2.** The DEAS events among three PTC variants.

|  | DEAS events |
| --- | --- |
| CPTC vs FPTC | NEDD4L_45649_AP, PTPN4_55141_AP, CRLF2_88358_AT, TNIP1_74126_AP, OXCT1_71882_AP, ACSL6_73248_AT, KIAA1217_10994_AP, LTBR_19846_AP, KLK10_51263_AP, LYNX1_85361_AP, RCAN2_76415_AP, TUBB3_38167_AP, PPARG_63413_AP, ARHGAP24_69814_AP, CTNNBIP1_578_AP, LYNX1_85366_AT, PADI4_851_AT, EPHA4_57746_AP, TNFRSF13B_39449_AT, ZNF575_50203_AP, TRAK1_64266_AP, RGS12_68637_AP, SAA2_14580_AT, LMF1_33029_AP, BID_61002_AP, HYAL3_64984_AP, TRIM46_7953_AT, MACROD2_58714_AP, ZNF331_51724_AP, DYSF_53937_ES, NAV1_9387_AP, ACSS2_59036_AP, NAV2_14696_AP, SERPINB5_45716_AT, SSPN_20842_AP, LRRC2_64456_AP, TUBB3_38166_AP, ELMO1_79266_AP, CD44_15130_ES, SHROOM4_89139_AP, NNMT_18817_AP, PLXNC1_23721_AP, SLA_85217_ES, AKT2_49869_AP, MAP2K6_43187_AP, COL14A1_85015_AP, DCN_23655_AT, TUBB3_38175_ES, NDE1_34181_AP, RAG1_15433_AT, RRM1_14033_AP, PI4K2A_12728_AP, SMTN_61810_AP, CD44_15111_ES, ST6GAL1_68067_AP, LAMB4_81393_AT, KIAA1217_10995_AP, FAM107A_65463_AP, MXRA8_142_AP |
| CPTC vs TCPTC | NNMT_18817_AP, MPP7_11093_ES, DCN_23655_AT, TMEM132D_25195_AP, C1S_20068_AP, RACGAP1_21625_AT, TUBB3_38175_ES, RTN4_53592_ES, KIAA1217_10995_AP, HMGA2_22877_AT, SSBP4_48427_AP, COL14A1_85015_AP, P2RY6_17682_AP, ELN_80044_ES, PAK6_29958_AP, RCAN2_76415_AP, UBN1_33869_AP |
| FPTC vs TCPTC | NEDD4L_45649_AP, RCAN2_76415_AP, ACSL6_73248_AT, TNIP1_74126_AP, ARHGAP24_69814_AP, CRLF2_88358_AT, CTNNBIP1_578_AP, PPARG_63413_AP, OXCT1_71882_AP, LYNX1_85361_AP, LTBR_19846_AP, LYNX1_85366_AT, SAA2_14580_AT, RGS12_68637_AP, KIAA1217_10994_AP, TUBB3_38167_AP, DYSF_53937_ES, ZNF575_50203_AP, TRIM46_7953_AT, KLK10_51263_AP, HDAC9_78886_AP, MAST1_47878_AT, PTPN4_55141_AP, TNFRSF13B_39449_AT, NAV1_9387_AP, TTC40_13507_AT, SERPINB5_45716_AT, NAV2_14696_AP, MKL1_62349_AP, ARHGAP22_11485_AP, PADI4_851_AT, HYAL3_64984_AP, ZNF331_51724_AP, LMF1_33029_AP, CORIN_69182_AT, PSG5_50182_AT, ACAP1_38921_AP, BTBD11_24189_AP, TRAK1_64266_AP, KCNAB1_67357_AP, ACSL5_13109_AP, ASPHD1_35984_AT, DNASE1L1_90575_AP, BID_61002_AP, EPHA10_1825_AT, FGF1_73869_AP, TANK_55731_AP, MGAT1_75022_AP, TMEM79_8217_AP, RAD23B_87147_AP, CREM_11230_AP, PSG5_50183_AT, SSPN_20842_AP, GPER1_78565_AP, ACSS2_59036_AP, SULT2B1_50773_AP, ITGA7_22216_ES, TSPEAR_60826_AT, SMAGP_21829_AP, DYSF_53934_AP, CKMT2_72660_ES, ATP2C1_66756_AP, EGFL7_88188_AP, IMMP1L_14816_ES, DIO2_28647_AP, MCC_73004_AP, BCAR3_3796_AP, CCDC108_57579_AT, ERAP2_72871_AD, EPHA4_57746_AP, SHROOM4_89138_AP, ANKRD30B_44762_AT, EVA1A_54148_AP, AHCYL2_81744_AP, KAZN_730_AP, RPS6KA1_1281_AP, SERPINA1_29130_ES, LPAR5_19900_AP, KLK10_51264_AP, TACC1_83437_AP, EXOC7_43568_ES, CCND3_76154_AP, BLOC1S6_30440_ES, ERMAP_2124_RI, TMC6_43757_AT, PPFIBP1_20893_ES, ACOX3_68765_AP, DST_76569_ES, NPIPB6_35703_AP, NARF_44391_AP, SS18_44966_ES, TMEM45B_19457_AP, C12orf73_24074_ES, CKMT1B_30276_AP, MEIS1_53805_AP, CACNB3_21480_ES, PIK3R1_72294_AP, RPS6KA3_88669_AP, SCNN1A_19842_ES, RARG_21980_AP, MCC_73006_AP, NCS1_87899_AP, TRIM16L_39631_AP, RTKN2_11871_AT, RAET1G_78131_AT, CBX5_22139_AP, CD44_15127_ES, NEDD4L_45651_AP, LMO7_26060_AP, BIN1_55184_ES, KCNC3_51170_AP, TIPARP_67366_AP, SPTBN1_53576_AP, FMO5_7367_AT, DYSF_53935_AP, KCTD13_35987_AT, RAG1_15433_AT, TAB3_88762_AA, GPR116_76428_AP, NALCN_26204_AT, GPR110_76437_AT, LCN6_88203_AP, MROH6_85424_AP, SULT2B1_50774_AP, EPB41L1_59264_AP, FGF1_73871_AP, CKMT1A_30304_AP, CD44_15130_ES, ELN_80043_AA, FAM86B1_82686_ES, TMEM132D_25195_AP, P2RY6_17681_AP, CCNDBP1_30220_ES, ST5_14263_AP, C2orf73_53572_AT, PDK1_55976_AT, ERBB3_22355_AT, SAA2_14577_AT, GLS2_22441_AA, NFYC_2015_AP, SALL1_36401_AP, ZNF581_52118_AP, ANK3_11843_AP, LARGE_61941_AP, DTNA_45092_AP, SLC1A2_15413_AT, ELP3_83202_AP, LYNX1_85363_AP, KAZN_729_AP, TMEM43_63521_ES, CTCFL_59903_AT, NAV1_9389_AP, PLXNC1_23721_AP, ASPHD1_35983_AT, ITGB4_43489_ES, ARHGAP24_69815_AP, SLC14A2_45328_AP, RTN4_53592_ES, APOC2_50375_RI, STRA6_31688_AD, TMEM79_8218_AP, MUC20_68183_AP, NAV2_14695_AP, F8_90666_AP, BRD3_88091_AA, VPS13D_706_AP, RACGAP1_21625_AT, TUBB3_38166_AP, SHROOM4_89139_AP, SMTN_61810_AP, AKT2_49869_AP, PDE1C_79184_AP, LAMB4_81393_AT, ELN_80045_ES, SSBP4_48427_AP, NDE1_34181_AP, SLC14A2_45330_AT, MAP2K6_43187_AP, P2RY6_17682_AP, PAK6_29958_AP, MPP7_11093_ES, ELN_80044_ES, ARHGEF10L_857_AP, ELMO1_79266_AP, HMGA2_22877_AT, LRRC2_64456_AP, FAM107A_65463_AP, SLA_85217_ES, GAREML_52882_AP, ST6GAL1_68067_AP, C1S_20068_AP, PI4K2A_12728_AP, RRM1_14033_AP, COL14A1_85015_AP, TUBB3_38175_ES, DCN_23655_AT, KIAA1217_10995_AP, NNMT_18817_AP, MXRA8_142_AP |

**Table S3.** The differences of DEAS events which were differentially expressed in PTC variants in clustering groups. P values were calculated by wilcoxon test.

| DEAS events | Cluster1/Cluster0 | | Cluster2/Cluster0 | | Cluster2/Cluster1 | |
| --- | --- | --- | --- | --- | --- | --- |
|  | log2FC | p value | log2FC | p value | log2FC | p value |
| TUBB3_38175_ES | 2.11 | 2.66E-42 | -1.43 | 1.00E-09 | -3.54 | 1.17E-21 |
| KIAA1217_10995_AP | 2.51 | 3.38E-54 | -0.79 | 7.46E-05 | -3.29 | 8.94E-22 |
| NNMT_18817_AP | 1.44 | 5.29E-11 | -2.93 | 2.84E-04 | -4.37 | 2.00E-11 |
| DCN_23655_AT | 1.59 | 1.87E-19 | -2.23 | 2.93E-13 | -3.83 | 6.64E-19 |
| RCAN2_76415_AP | -1.24 | 3.02E-10 | 2.53 | 5.50E-26 | 3.77 | 2.89E-22 |
| COL14A1_85105_AP | 1.12 | 4.15E-10 | -1.20 | 0.0190 | -2.32 | 1.61E-08 |

**Table S4.** Three bioactive compounds with common mode of action in the CMap analysis.

| Name | Score | Description | Target | MOA |
| --- | --- | --- | --- | --- |
| Orantinib | -99.79 | FGFR inhibitor | PDGFRB, AURKA, AURKB, KDR, EGFR, FGFR1, FGFR2, PDGFRA, TBK1 | FGFR inhibitor, VEGFR inhibitor, PDGFR receptor inhibitor |
| Tyrphostin-AG-1295 | -99.47 | PDGFR receptor inhibitor | FLT3, KDR, PDGFRA, PDGFRB | PDGFR receptor inhibitor |
| AG-370 | -99.47 | PDGFR receptor inhibitor | PDGFRB | PDGFR receptor inhibitor |
